# Compressing the collective knowledge of ESM into a single protein language model

**DOI:** 10.1038/s41592-026-03050-9

**Published:** 2026-03-30

**Authors:** Tuan Dinh, Seon-Kyeong Jang, Noah Zaitlen, Vasilis Ntranos

**Affiliations:** 1 Department of Epidemiology and Biostatistics, University of California, San Francisco, San Francisco, CA USA; 2 Department of Computational Medicine, David Geffen School of Medicine, University of California, Los Angeles, Los Angeles, CA USA; 3 Department of Human Genetics, David Geffen School of Medicine, University of California, Los Angeles, Los Angeles, CA USA; 4 Department of Neurology, David Geffen School of Medicine, University of California, Los Angeles, Los Angeles, CA USA; 5 Institute for Human Genetics, University of California, San Francisco, San Francisco, CA USA; 6 Diabetes Center, University of California, San Francisco, San Francisco, CA USA; 7 Bakar Computational Health Sciences Institute, University of California, San Francisco, San Francisco, CA USA

**Keywords:** Machine learning, Protein function predictions, Computational models, Genetics

## Abstract

Protein language models (PLMs) have recently emerged as a promising approach for next-generation variant-effect prediction (VEP). Most high-performing VEP methods currently utilize PLMs combined with additional information, such as homology, protein structure and population genetics data to improve prediction accuracy. This performance gain, however, comes with added complexity or limited applicability compared to pure PLMs trained only on raw, unaligned sequences, such as evolutionary scale modeling (ESM). Here we challenge the prevailing view that sequence-only PLMs are intrinsically limited and present an efficient co-distillation approach to adapt them for high-accuracy VEP without requiring additional information beyond evolutionary signals captured during pretraining. We allow individual PLMs to self-improve by distilling the most confident predictions from multiple models of the same family and demonstrate that co-distillation of ESM models suffices to achieve state-of-the-art performance across multiple VEP benchmarks. We further show that this performance increase enables accurate quantification of the severity of variant effects on continuous clinical phenotypes in biobank data.

## Abstract (web summary)

A co-distillation framework is used to iteratively adapt sequence-only protein language models for high-accuracy variant effect prediction, without the need for additional structural or genetic data. Individual protein language models therefore self-improve by distilling the most confident predictions from multiple models, achieving state-of-the-art performance across multiple variant effect prediction benchmarks.

## Main

Predicting the functional consequences of genetic variants, known as variant effect prediction (VEP), is a fundamental computational challenge with important applications in human genetics, drug development and protein engineering^1–5^. In recent years, the field of VEP has seen substantial advancements, particularly with the emergence of protein language models (PLMs)^6–13^. These models, inspired by transformative developments in natural language processing, are trained on vast repositories of protein sequences^8^ to capture the intricate patterns and relationships within the protein sequence space, demonstrating remarkable capabilities in VEP and other protein-related tasks, including protein structure prediction^9,14^, function annotation^15^ and protein design^16^.

To further increase predictive performance, VEP methods often employ a hybrid approach, combining PLMs with additional sources of information such as multiple sequence alignments (MSA), protein structures and population genetics data. Indeed, the top-performing methods on the ProteinGym Deep Mutational Scan (DMS) benchmark^17^, including Saprot^14^, TranceptEVE^18^ and others^19,20^, are all PLMs that have been trained on three-dimensional (3D) structure or MSA data and use this information during inference to make accurate predictions of variant effects. The recently developed closed-source models PrimateAI-3D^13^ and AlphaMissense^7^ also follow a hybrid approach, demonstrating similar performance gains by integrating both modalities with additional fine-tuning on human and primate population genetics data. Additionally, in theory, protein sequence information alone should be sufficient to achieve the same level of accuracy, at the time of testing, sequence-only PLMs are substantially underperforming compared to these more recent approaches.

Although effective, these hybrid approaches come with increased complexity and may have limited applicability compared to sequence-only PLMs in scenarios where such additional data are unavailable, incomplete or computationally expensive to obtain. Moreover, the integration of diverse data sources can introduce biases and dependencies that may affect both the interpretability and generalizability of the predictions in downstream tasks. Experimental 3D structures, for example, are only available for a small fraction of proteins, algorithmic and hyperparameter choices can have variable effects on MSA quality, and the use of population genetics data can lead to data circularity concerns in clinical genetics applications. Even though having this extra information alongside the sequence can certainly help on average, it is not clear how each modality contributes to each prediction and, more importantly, how reliable the corresponding model can be in its absence. The performance of the sequence-only PLM versions of TranceptEVE and SaProt, for example, in the absence of MSA and 3D structure decreases substantially, indicating a strong dependence on the availability of these additional modalities^14^. By contrast, pure evolutionary scale PLMs, trained solely on unaligned protein sequences, have the potential to offer a more balanced, streamlined and broadly applicable approach to VEP.

In this work, we focus on the widely adopted, sequence-only PLMs of the evolutionary scale modeling (ESM) family^8,9,21^ and show that their performance is not fundamentally limited compared to the above more complex modeling approaches. In particular, we demonstrate that better detection of the evolutionary signals captured during the pretraining of different ESM models has the capacity to substantially increase the VEP performance of all individual models without using any external information. To achieve this, we introduce a co-distillation framework within which multiple models can learn from each other, alternating their role as teachers and students depending on their estimated confidence for each prediction; the log-likelihood ratio (LLR) of the model that provides the most confident prediction for any given mutation is used to refine the predictions of the others. Through comprehensive evaluation across multiple VEP benchmarks, we demonstrate that our approach produces substantially improved variant-effect ESM models (VESM) that match or surpass current state-of-the-art VEP methods, effectively closing the performance gap between hybrid and sequence-only PLMs. We further extend this framework to incorporate structural information in a modular fashion, preserving VESM’s advantages while improving performance on structure-dependent tasks. Finally, we demonstrate our models’ capacity to accurately quantify the continuous effect of variants on clinical phenotypes in large-scale population studies, extending their utility from binary classification to quantitative trait prediction in clinical genetics.

## Results

### A maximum confidence strategy for detecting evolutionary signals across multiple PLMs

**Fig. 1:**
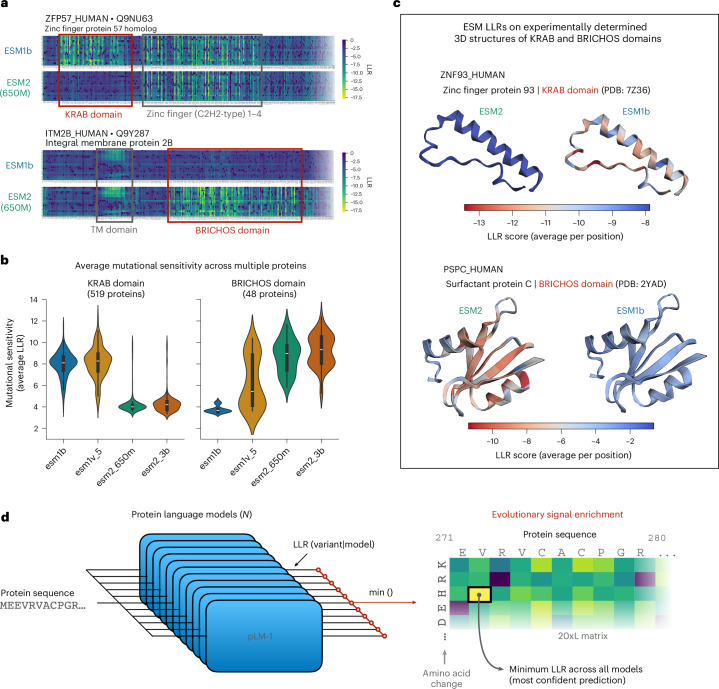
Enriching for evolutionary signals by searching across multiple PLMs. **a**, An example of ESM2 and ESM1b complementarity in detecting evolutionary conserved domains as mutationally sensitive; the 20xL heat map of all LLR scores obtained from each model is shown for two proteins (ZFP57 and ITM2B) that are involved in human disease. ESM1b is able to correctly identify the majority of mutations within the annotated KRAB domain in ZFP57 as damaging (yellow color), whereas ESM2 predicts them as neutral (blue color). The opposite is true for the BRICHOS domain in ITM2B. **b**, An evaluation of the capacity of different ESM models in detecting these domains across multiple proteins (*n* = 519 and *n* = 48 proteins with annotated KRAB and BRICHOS domains, respectively). The average score of all possible mutations that fall within each domain is calculated for each protein as a proxy for each model’s ability to detect it. The violin plot shows these average scores across all proteins for each model (median, center line; interquartile range, box; whiskers, 1.5× interquartile range). **c**, The average LLR per position as predicted by ESM2 and ESM1b is visualized for two other proteins (ZNF93 and PSPC) with experimentally determined structures of the two domains (PDB: 7Z36 and 2YAD). **d**, An illustration of the maximum confidence approach; signal‑enriched data are generated for all possible mutations by combining predictions from *N* models. Individual mutations are scored by choosing the most confident prediction (minimum LLR; indicated by ‘min()’) across all models (Methods).

A PLM tries to learn an approximate evolutionary fitness landscape by capturing evolutionary patterns across the millions of unaligned sequences it has seen during pretraining. This is an incredibly challenging task, and although the resulting models are surprisingly effective in identifying conserved motifs, domains and other co-evolution signals, they can also have certain blind spots. For example, in Fig. 1, we show that ESM2 models (independent of parameter size) consistently fail to identify conserved KRAB domains as annotated within 519 proteins on Uniprot, whereas ESM1b and ESM1v (both 650-million (hereafter denoted as 650M)-parameter models) consistently fail to identify the less common but well conserved BRICHOS domains as annotated within 48 proteins. For each domain, however, there exists at least one model that has detected the corresponding motifs and can correctly identify them as conserved mutationally sensitive regions within the protein. This large effect is quite unexpected as all of these models, members of the ESM family, have almost identical architecture and were trained on similar data^8,9,21^. In the context of predicting the pathogenicity of variants, failure to detect these signals would lead to an increased number of false-negative errors for each model. Yet the information for detecting both domains is captured within the model family.

Here, we first consider a simple approach that can exploit this complementarity and enrich for evolutionary signals captured during the pretraining of multiple PLMs. In particular, we use all individual models to calculate the LLRs of all possible missense mutations for a given wild-type (WT) sequence and aggregate their predictions at the variant level by taking the minimum (ESMIN; Fig. 1d). This approach effectively scores each variant by choosing the model that outputs the minimum LLR or, equivalently, has the maximum confidence in the WT residue compared to the mutated amino acid (Methods). Intuitively, this is most effective when different models have varying strengths in predicting mutations in different protein contexts, as demonstrated in the above examples. We contrast this with the more commonly used averaging approach, which can potentially dilute subtle evolutionary signals detected only by a small fraction of the models.

**Extended Data Fig. 1:**
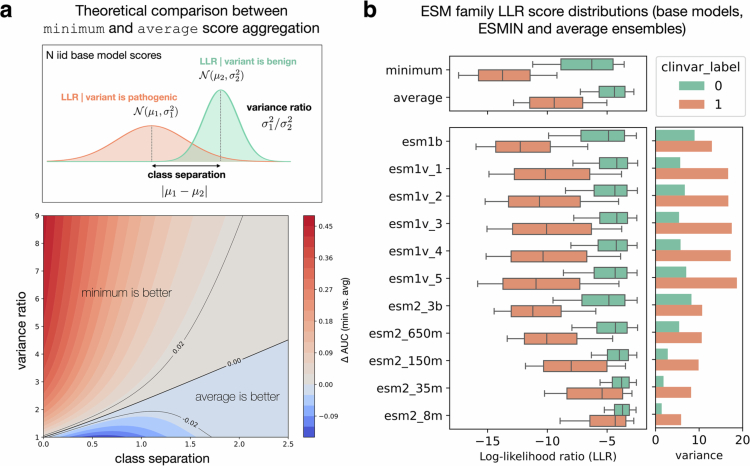
Theoretical comparison of minimum vs average aggregation. (**a**) Schematic of the Gaussian model used to compare aggregation strategies: class-conditional LLR distributions with means (μ) and variances (σ^2^), and the resulting AUC landscape as a function of class separation |μ_1_-μ_2_| and variance ratio σ_1_^2^/σ_2_^2^. Regions favoring minimum-LLR aggregation (max-confidence) are shaded accordingly. (**b**) Empirical ClinVar LLR distributions for individual ESM models (benign vs pathogenic), compared with the minimum (ESMIN) and average ensembles; the right panel shows per-class variances. The pathogenic class exhibits larger dispersion, aligning with the regime in which minimum aggregation is expected to outperform averaging.

To make this intuition precise, we develop a simple theoretical framework (Supplementary Note 1) in which, conditional on the true class (pathogenic versus benign), the LLR scores generated by *N* base models are independently drawn from a Gaussian distribution. This mathematical model is governed by two interpretable quantities: the separation between the class means and the ratio of their variances (Extended Data Fig. 1a). For both the minimum (maximum confidence) and the averaging aggregators, we derive analytical expressions for the area under the receiver operating characteristic (ROC) curve (area under the curve (AUC)) and evaluate them numerically to map the regimes in which each strategy performs best. In this independent and identically distributed (i.i.d.) setting, we show that minimum aggregation can substantially outperform averaging when pathogenic LLRs are much more dispersed than the benign LLRs. Empirically, we observe that ESM models exhibit this property (Extended Data Fig. 1b). Although ESM models are not independent, our analysis clarifies how maximum confidence can exploit their variance asymmetry to better detect and amplify evolutionary signals captured within the family.

**Extended Data Fig. 2:**
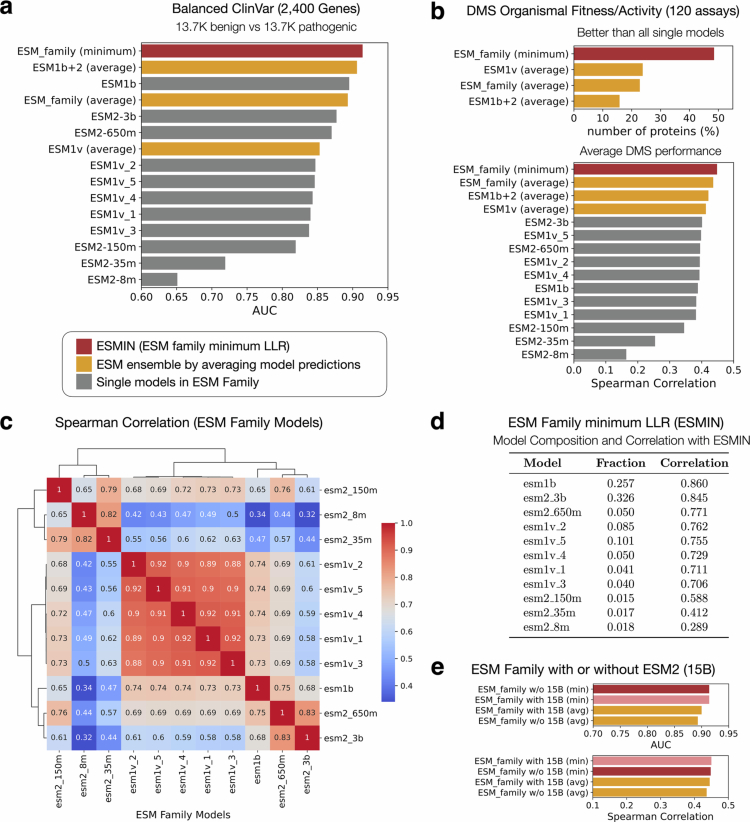
The ESM Family of models. (**a**) Performance of ESMIN on ClinVar (2,400 genes with a total of ~27k pathogenic and benign variants, class balanced per gene) compared to other ESM family ensembles and individual models. ESM_Family is composed of the 11 ESM models contributing to ESMIN. ESM1b + 2 is the equal-weight average of the ESM1b and ESM2-650M models and ESM1v is the ensemble of the 5 individual ESM1v models. (**b**) Performance evaluation on ProteinGym (120 organismal fitness and activity DMS assays, across 101 proteins). The top right panel shows the percentage of DMS assays for which an ESM ensemble outperforms all individual ESM family models. (**c**) Heatmap of Spearman correlations between ESM model predictions that contribute to ESMIN. (**d**) The fraction of model predictions included in ESMIN and the Spearman correlation with ESMIN are shown for each model. (**e**) Effect of adding ESM2-15B to the family; ESMIN and average ensemble performance on ClinVar (top) and ProteinGym DMS (bottom), showing negligible additional gains from including ESM2-15B.

To evaluate the efficacy of the maximum confidence strategy in a VEP setting, we aggregated proteome-wide predictions from all individual, sequence-only models within the ESM family (ESM1b, five ESM1v models and five ESM2 models, excluding only the largest 15-billion-parameter model) and benchmarked the resulting predictions (ESMIN) on both Clinical and DMS^22^ data (Extended Data Fig. 2). To address concerns that ESMIN might indiscriminately predict all variants of the same gene to be pathogenic, we specifically assessed its performance on a per gene class-balanced version of the ClinVar dataset (Methods). We also investigated the correlation structure within the ESM family and how much each individual model contributes to ESMIN (Extended Data Fig. 2c,d). The models cluster into three groups by prediction similarity, with ESM1b and ESM2-3B contributing the most to ESMIN (25.7% and 32.6%). ESM2-650M contributes only 5%, but it is highly correlated with ESMIN scores, probably due to its similarity with ESM2-3B. Further, including the much larger ESM2-15B model when calculating ESMIN scores does not seem to provide additional performance gains compared to the 11 model ESM family ensemble (Extended Data Fig. 2e).

Our results show that ESMIN surpasses alternative ensemble methods, including averaging the predictions of all models, averaging the predictions of the best two models as well as the ESM1v ensemble that was specifically developed for VEP. This extends to the DMS benchmark from ProteinGym, where ESMIN achieves a higher average Spearman correlation between model predictions and experimental measurements across 120 organismal fitness and activity assays. Importantly, ESMIN achieves better performance than all individual ESM models in a much larger fraction (~50%) of the DMS assays compared to other ensembles (~20%), indicating that maximum confidence benefits multiple proteins and can be potentially used to provide strong training signals for improving the performance of ESM models.

### Maximum confidence co-distillation of ESM models improves their performance in VEP and downstream tasks

**Fig. 2:**
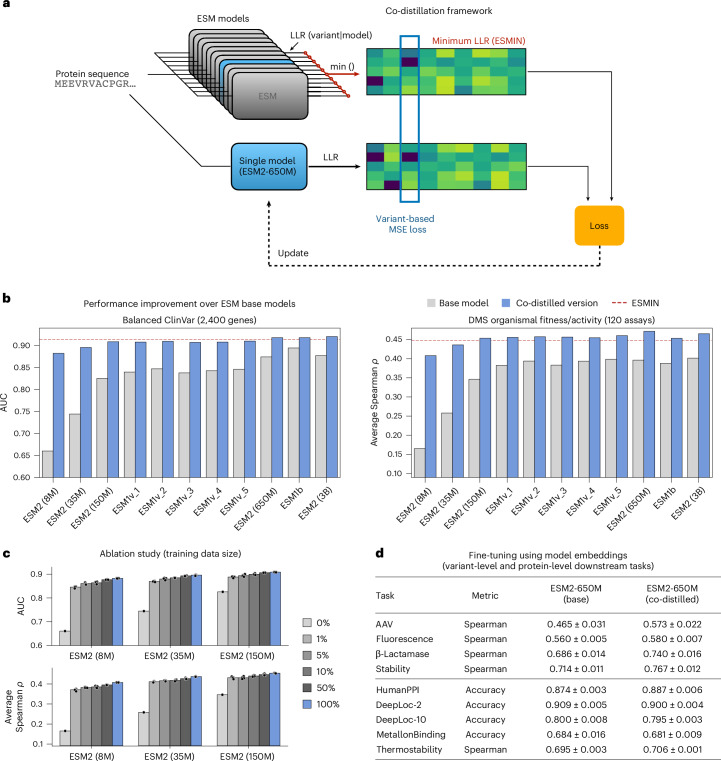
Maximum confidence co-distillation of the ESM family substantially improves individual ESM models. **a**, An overview of the co-distillation framework; for each protein, all models make predictions, which are then combined into a single 20xL matrix by taking the element-wise minimum (maximum confidence per variant). Each individual model is then updated by calculating a loss with respect to its own output (Methods). **b**, A performance evaluation of individual co-distilled models compared to their baseline on ClinVar (2,400 genes with a total of ~27K pathogenic and benign variants, class balanced per gene (left)) and ProteinGym (120 organismal fitness and activity DMS assays (right)). The dashed red lines indicate the performance of ESMIN. **c**, An ablation study varying the percentage of proteins used as training data during co-distillation. The full dataset (100%, including the validation split) contains 18,683 human proteins. Ablations were made by first excluding all proteins that are similar to the ones used in the benchmark (5,279 proteins, >30% sequence similarity) and then sequentially eliminating proteins from the remaining set (nested subsets). **d**, The performance of learned model embeddings in nine downstream tasks (supervised fine-tuning on experimental measurements). Task-independent (frozen) embeddings were extracted from the base and co-distilled versions of ESM2-650M and used to train task-specific models (Methods). The table reports the mean and s.d. across three random seeds; statistically significant differences (two-sided paired *t*-test; *P* < 0.05) are highlighted in bold.

Although leveraging the most confident model for scoring individual mutations enhances VEP performance, it introduces practical challenges and limitations compared to using a single PLM. The collective parameter count of all models contributing to ESMIN, for example, exceeds 7 billion, making it computationally prohibitive for large-scale VEP. Furthermore, the multimodel approach complicates the use of embeddings for supervised fine-tuning on downstream tasks, which is a key application of PLMs. To overcome these limitations, we develop a co-distillation framework that uses the same maximum confidence approach within the ESM family to collectively improve the performance of individual ESM models (Fig. 2a).

Our proposed framework uses the principle of mutual learning, where models serve as both teachers and students, depending on their confidence for each prediction. For any given mutation, the most confident prediction (ESMIN) is used to guide the learning of the other models. We applied this framework to the ESM family and trained improved versions of all ESM models up to 3 billion parameters. The training process is efficient and can be performed independently for each individual model, using precomputed data (Methods).

**Extended Data Fig. 3:**
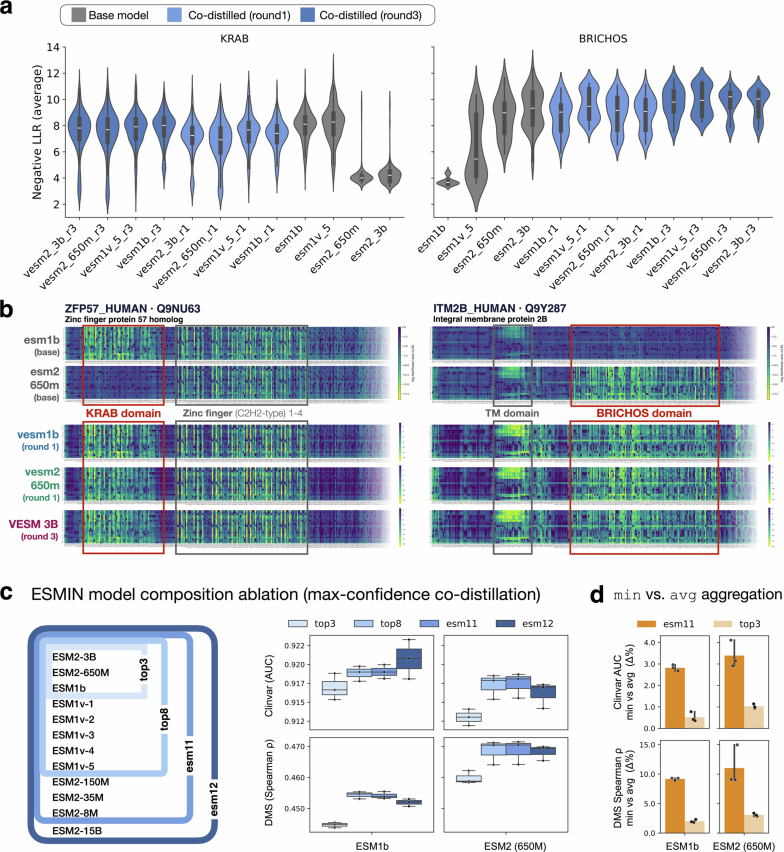
Max confidence co-distillation. (**a**) Recovery of conserved domain signals after co-distillation. For KRAB (n = 519 proteins) and BRICHOS (n = 48), the violin plots (median, centre line; interquartile range, box; whiskers, 1.5× IQR) show the average LLR (mutational sensitivity) across domain positions for base models versus their co-distilled counterparts (round 1 denoted as _r1). Final round co-distilled models (denoted as _r3) are also included for reference. All co-distilled models are able to recover signals missed by their own bases (see Fig. 1b). (**b**) LLR heatmaps for ZFP57 (KRAB) and ITM2B (BRICHOS); while base ESM1b and ESM2-650M models miss one domain each (as highlighted in Fig. 1a), their co-distilled versions can identify both. (**c**) Model composition ablation for max-confidence co-distillation (round-1) using only the top-3, the top-8, the default 11-model set, or all 12 (incl. 15B). Performance on Balanced ClinVar and DMS fitness/activity shows that performance saturates once a moderately diverse medium-to-large subset is included. (**d**) Minimum (max-confidence) versus average aggregation under the top-3 and 11-model settings. Minimum aggregation yields consistent performance gains, especially with more diverse model sets.

The results of our ESM co-distillation were striking, with all models showing remarkable improvement (Fig. 2b). Notably, we observed unexpectedly large gains in low-capacity ESM2 models (with 8M and 35M parameters). For instance, the AUC of ESM2-8M on the ClinVar dataset improved dramatically from 65% to nearly 88%. Remarkably, the co-distilled versions of ESM1b, ESM2-650M and ESM2-3B not only improved but surpassed ESMIN’s performance in both benchmarks, a phenomenon akin to the ‘student surpassing the teacher’ effect observed in deep learning and knowledge distillation literature^23,24^. With respect to the examples highlighted in Fig. 1, we saw that co-distilled models were now able to detect both domains as mutationally sensitive, effectively learning from each other (Extended Data Fig. 3a,b).

To assess the generalization capability of the co-distilled models and rule out simple memorization of ESMIN, we further conducted an ablation study where we varied the percentage of proteins used as training data during co-distillation (Fig. 2c; Methods). In particular we exclude from the pool of training data all human proteins that share more than 30% sequence similarity to any protein that is contained in the two benchmarks (2,400 proteins in Balanced ClinVar and 120 proteins in DMS organismal fitness and activity assays). Overall, this step excluded 5,279 sequences from the full training dataset of 18,683 human proteins. We then randomly eliminated proteins from this reduced set, creating nested subsets of decreasing size (50%, 10%, 5% and 1% of all sequences), providing a stringent control against overfitting to the human proteome. Our results show that co-distillation is very effective even with limited data, providing a very sharp increase in performance for all models considered in this setting, consistently across all five random seeds used for subsampling the training set. For example, co-distillation with only 1% of the human proteins (~200 sequences), already allows ESM2-35M to achieve 97% and 94% of its peak performance in the Balanced ClinVar and DMS benchmarks, respectively (AUC of 0.869/0.896 and *ρ* = 0.411/0.437).

In addition to the training-size ablation, we further examined how the choice of participating models affects co-distillation (Extended Data Fig. 3c). Specifically, we evaluated three additional co-distillation configurations with (1) only the top-3 performing ESM models within the family (ESM2-3B, ESM2-650M and ESM1b), (2) a top-8 subset (excluding the three smallest models) and (3) all 12 ESM models (including ESM2-15B). Relative to the 11-model configuration used in our main results, performance only dropped noticeably for the top-3 subset. By contrast, both the top-8 and full 12-model configurations matched the 11-model performance within error across benchmarks, indicating that most of the benefit is retained once a moderately diverse set of medium-to-large models is included. This is in line with our previous observation that minimum aggregation alone does not benefit from the inclusion of the ESM2-15B model (ESMIN; Extended Data Fig. 2e). Finally, we compared maximum-confidence co-distillation (using minimum aggregation) with average-aggregation co-distillation for the top-3 and 11-model settings (Extended Data Fig. 3d). Consistent with our earlier analysis of aggregation independent of co-distillation (Extended Data Fig. 2a,b), the maximum-confidence approach outperformed averaging in both settings, albeit with a much smaller margin in the three-model case (for example, ~10% versus ~2.5% improvement in DMS Spearman correlation for the 11-model and top-3 configurations, respectively).

Finally, we evaluated whether our co-distillation framework produces models with improved protein embeddings, which are widely used as sequence representations for fine-tuning on downstream tasks^25–32^. This is important to further assess the broader scope of our co-distilled models and their capacity to serve as improved versions of the corresponding base models, since introducing new knowledge to pretrained models can have the opposite effect and lead to catastrophic interference^33^, where updated models forget previously learned information. To test this in the context of protein representation learning, we extracted task-independent (frozen) embeddings from both the base and co-distilled versions of ESM2-650M and used them to train task-specific models for nine different downstream tasks involving supervised fine-tuning on various experimental measurements and protein annotations (Fig. 2d; Methods). Our results demonstrate that the co-distilled model embeddings yield substantial gains on all variant-level tasks (adeno-associated virus, fluorescence, β-lactamase and stability), without compromising performance when fine-tuned on protein-level tasks (for example, protein localization; DeepLoc).

### Iterative co-distillation converges to a single high-performance model

**Fig. 3:**
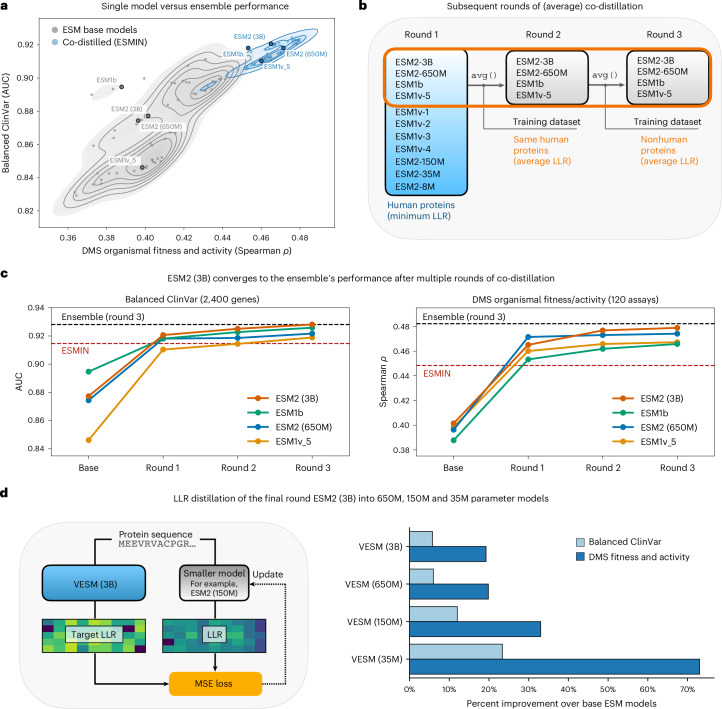
Iterative averaging co-distillation effectively compresses the ESM family into a single PLM. **a**, Single model versus ensemble performance on Balanced ClinVar and ProteinGym DMS benchmarks. Each point represents a pairwise ensemble obtained by averaging the predictions of two individual models (base ESM ensembles in gray, co-distilled ensembles in blue). Contour lines indicate performance density. Single model performances are annotated for reference. **b**, An overview of the subsequent rounds of averaging co-distillation. **c**, The performance across co-distillation rounds for the four participating models on Balanced ClinVar (left) and ProteinGym DMS (right). All models show monotonic improvement across rounds, with ESM2-3B converging to match the ensemble’s performance (dashed line) on both benchmarks after round 3, yielding VESM-3B. **d**, LLR distillation of VESM-3B into smaller-parameter models. Left: an overview of the knowledge distillation process (Methods). Right: percent improvement of the resulting VESM models over their corresponding base ESM models across both benchmarks.

Although co-distillation substantially improved individual ESM models, we found that additional performance gains were still achievable. One might expect that models co-distilled on the same training signals would converge to similar predictions, eliminating the benefit of aggregation. However, averaging predictions from the co-distilled models still yielded ensembles that outperformed their constituent single models on both Balanced ClinVar and ProteinGym DMS benchmarks (Fig. 3a). Both minimum and average aggregation strategies produced ensembles superior to individual models, with averaging providing a small but consistent advantage over minimum aggregation (0.048% AUC, 95% confidence intervals (CI) 0.016–0.081; 0.692% Spearman’s *ρ*, 95% CI 0.41–0.97). This shift from minimum to average aggregation as the superior strategy aligns with our theoretical analysis: co-distillation increases class separation while making the class-conditional dispersions more comparable between pathogenic and benign variants (Extended Data Fig. 1). In this regime, averaging is favored over minimum aggregation, which no longer benefits from asymmetric variance.

Guided by these observations, we investigated whether additional rounds of averaging co-distillation could further improve performance and ultimately converge to a single model capturing the full ensemble’s capabilities. We selected the top four co-distilled models from the first round (ESM2-3B, ESM1b, ESM2-650M and ESM1v5) and performed two additional rounds of averaging co-distillation (rounds 2 and 3; Fig. 3b). To enhance learning capacity, we increased the number of trainable parameters for all models and expanded the training set to include nonhuman sequences in the third round (Methods). Performance improved monotonically across rounds for all four models, with the largest model (ESM2-3B) matching the ensemble’s performance on both benchmarks (Fig. 3c). We refer to this converged model as VESM-3B.

**Extended Data Fig. 4:**
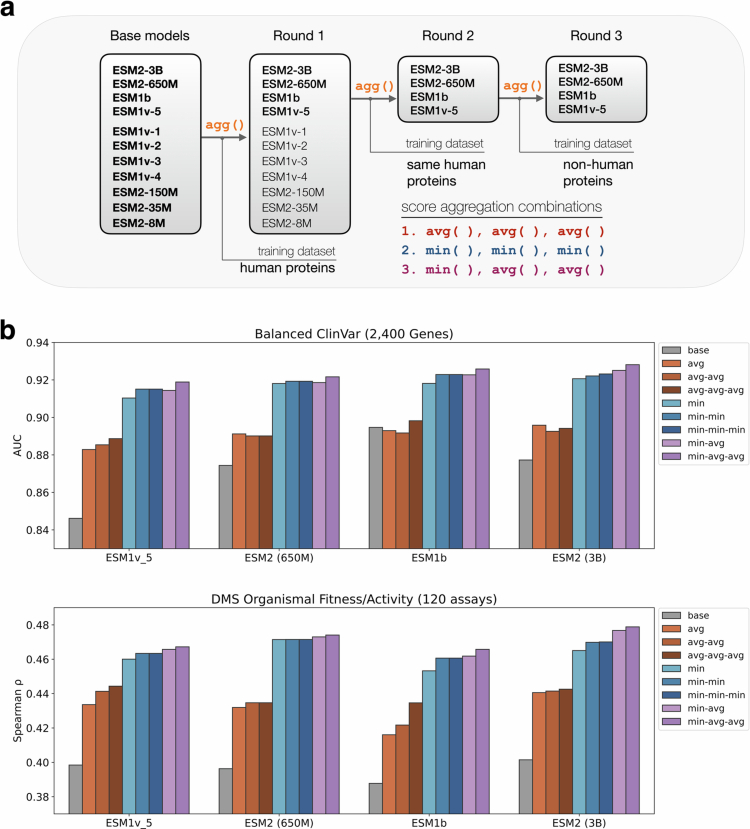
Aggregation strategy ablation across all co-distillation rounds. (**a**) Schematic of three co-distillation rounds and the aggregation schedules tested: *avg–avg–avg, min–min–min*, and *min–avg–avg*. Round 1 uses human proteins; round 2 repeats on the same set; round 3 uses non-human proteins (see Methods). (**b**) Performance on Balanced ClinVar (top) and ProteinGym DMS (bottom) for all four participating models (ESM1v-5, ESM2-650 M, ESM1b, ESM2-3B) across schedules. Using minimum aggregation in round 1 is required to achieve high performance; both aggregation strategies yield similar gains in subsequent rounds after minimum aggregation is employed in the first round.

We also tested how alternative methods of aggregation perform across all three rounds of co-distillation, ablating the use of minimum aggregation during the first round (maximum-confidence round) as well as the use of averaging in the last two (Extended Data Fig. 4). Overall, our evaluation across both benchmarks showed that maximum-confidence co-distillation in the first round is an essential step to achieve high performance in later rounds. After the first round, the choice between averaging and minimum aggregation had a much smaller but noticeable effect in final model performance.

To create a family of VESM models across different parameter scales and make them broadly accessible across compute budgets, we further distilled VESM-3B into smaller 650M, 150M and 35M parameter models (Fig. 3d; Methods). All distilled VESM models substantially outperformed the base ESM models and achieved comparable performance to VESM-3B (>98% and >93% on Balanced ClinVar and ProteinGym DMS, respectively). The improvements were particularly striking for smaller models, with VESM-35M improving performance by more than 20% on ClinVar and more than 70% on DMS compared to the original ESM2-35M (Fig. 3d, right).

### Sequence-only VESM models outperform state-of-the-art methods in predicting the clinical impact of variants

Co-distillation of ESM2-3B alongside the rest of the ESM family models produced VESM-3B, which is a single, 3-billion-parameter PLM, effectively trained only on unaligned (UniRef^34^) sequences. This model was able to surpass the collective performance of the ESM family, regardless of how individual model predictions were combined (maximum-confidence, average of all models, ESM1v, average of ESM1b and ESM2-650M), effectively achieving a ~61% reduction in total number of parameters compared to the full ensemble. Smaller models distilled directly from VESM-3B, maintained comparable performance with only 8%, 2% and 0.5% of the parameters. Here, we explore how these models compare with state-of-the-art VEP methods and hybrid approaches that further require the use of MSA or 3D structure information to enhance VEP performance.

**Fig. 4:**
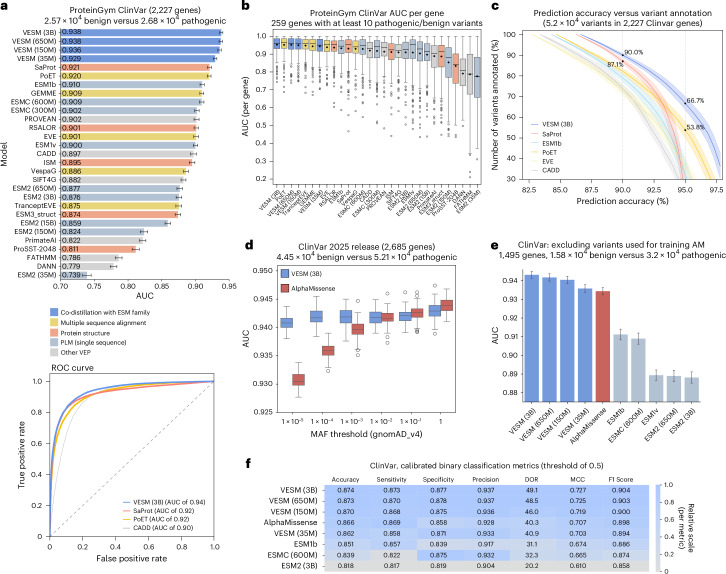
Performance evaluation of sequence-only VESM models on clinical VEP. **a**, Benchmarking of VESM models against 24 other PLMs and VEP methods on an independent, publicly available ClinVar benchmark from ProteinGym (2.57 × 10^4^ benign and 2.68 × 10^4^ pathogenic variants across 2,227 genes). Models are color-coded based on the additional sources of information they use. Top: global AUC is shown for each model. Error bars correspond to s.d. of the ROC-AUC scores centered around the mean (estimated by bootstrapping; *n* = 50 label-balanced resamples of 6,000 variants). Bottom: the corresponding ROC curve is shown for top-performing models of each class. **b**, A boxplot of the ClinVar AUC per gene for *n* = 259 genes that have at least ten pathogenic or benign labeled variants. **c**, The percentage of ClinVar variants that can be confidently classified by each model. The prediction accuracy and the total number of annotated variants are computed by varying the classification threshold for each method. The average and a two-standard-deviation interval across *n* = 10 bootstraps are shown (Methods). **d**, A comparison with AlphaMissense^7^ on a recent (03/25) release of the ClinVar dataset. A boxplot of the AUC achieved by each model across a range of MAF filtering thresholds from gnomAD v4 (Methods). **e**, A performance evaluation on a subset of the ClinVar dataset that excludes all human variants that have been used for training AlphaMissense (MAF >1×10^−5^ in gnomAD v2). The error bars correspond to s.d. of the ROC-AUC scores centered around the mean (estimated by bootstrapping; *n* = 100 label-balanced resamples of 10,000 variants). **f**, Calibrated binary classification metrics evaluating the performance of each model on the same MAF-filtered ClinVar dataset used in **e**. The boxplots show: center line (median); box limits (Q1, Q3); whiskers (±1.5× interquartile range); points (outliers).

To provide an objective assessment of the models’ capabilities, we evaluate clinical VEP performance on an independent, publicly available ClinVar benchmark obtained from ProteinGym^17^ curating 2.57 × 10^4^ benign and 2.68 × 10^4^ pathogenic variants across 2,227 disease associated genes (Fig. 4). Overall, we compare our VESM models with 24 other methods including sequence-only PLMs, recently developed hybrid PLMs trained on protein structure (for example, Saprot^14^, ESM3^35^ or ProSST^19^) or MSA information (for example, PoET^20^, EVE^36^, TranceptEVE^18^, GEMME^37^ or VespaG^38^), as well as other widely used VEP methods that leverage homology-based (for example, SIFT^39^ or PROVEAN^40^) or population-based (for example, PrimateAI^13,41^ or CADD^42^) approaches (Methods).

Surprisingly, our results demonstrate that all sequence-only VESM models performed exceedingly well on ClinVar, using both global and per-gene average AUC as the evaluation metric, effectively closing the performance gap to more complex VEP methods (Fig. 4a,b). Notably, all VESM models showed substantial performance gains over all other sequence-only PLMs, including the recently released ESM-C^43^. We also evaluated the entire ROC curve for the top-performing models of each class (VESM-3B, sequence only; SaProt, structure based; PoET, alignment based; and CADD, population based). We observe that even though SaProt is matching VESM-3B performance in the low false positive regime (high specificity), it is not able to maintain a similarly high true positive rate for the rest of the curve (high sensitivity regime). The opposite holds for CADD and PoET. VESM-3B on the other hand is more balanced, maintaining high performance across the entire ROC.

**Extended Data Fig. 5:**
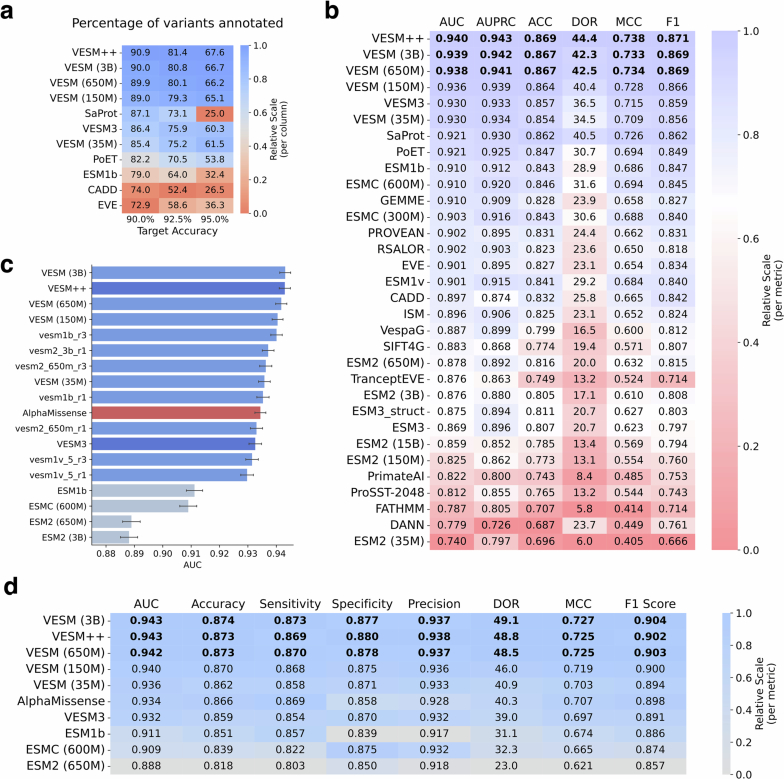
Expanded evaluation of all VESM and baseline models on ClinVar. (**a**) ClinVar annotation coverage versus prediction accuracy shows the percentage of variants confidently classified by each method (benign or pathogenic) at varying accuracy levels. (**b**) Performance evaluation using calibrated binary classification metrics (accuracy, diagnostic odds ratio, MCC, and F1-score) at a fixed threshold of 0.5. Threshold-independent AUC and AUPRC are shown for reference. (**c**) Performance evaluation on a subset of the ClinVar dataset that excludes all human variants that have been used for training AlphaMissense (MAF>1e-5 in gnomAD v2). Error bars correspond to the s.d. of the ROC-AUC scores centered around the mean (estimated by bootstrapping; n = 100). (**d**) Calibrated binary classification metrics evaluating the performance of each model on the same MAF-filtered ClinVar dataset. All panels shown here are related to the main results shown in Fig. 4.

We also evaluated how VESM-3B compares with other unsupervised methods in a clinical annotation task. In particular, we computed a curve that measures the percentage of ClinVar variants that each method can confidently annotate (either as benign or pathogenic), as a function of prediction accuracy (Fig. 4c and Extended Data Fig. 5a; Methods). We see that VESM-3B consistently outperforms the other methods, maintaining the highest annotation coverage across all accuracy levels. Specifically, VESM-3B annotations at 90% accuracy can cover 90% of ClinVar, which is a substantial improvement from 79% achieved by ESM1b, the currently best-performing sequence-only PLM. Moreover, at this level of accuracy, the second-best method, SaProt (87% coverage), performs substantially better than the other two homology-based methods, PoET (81%) and EVE (73%). At 95% accuracy, a level of accuracy that SaProt could achieve for only 25% of the variants, VESM-3B is still able to annotate ~67%, compared to 54% achieved by PoET. The binary classification performance of all methods is further evaluated across multiple metrics in Extended Data Fig. 5b using a fixed threshold of 0.5. All VESM models performed exceptionally well on this benchmark, with VESM-3B and VESM-650M surpassing all other non-VESM methods across all evaluated metrics (area under the ROC, area under the precision-recall curve, accuracy, diagnostic odds ratio (DOR), Mathews correlation coefficient (MCC) and F1 score).

Finally, we provide a direct comparison between VESM-3B and AlphaMissense^7^, a state-of-the-art closed-source VEP model. AlphaMissense is an ensemble model building on top of the AlphaFold architecture, trained on 3D protein structure and MSA information. Beyond these two modalities, AlphaMissense is further trained using human and primate population allele frequency information, enabling state-of-the-art variant pathogenicity classification performance. However, this introduces data circularity with respect to ClinVar labels, because the allele frequency of a variant is considered as strong supporting evidence contributing to its clinical annotation (ACMG guidelines^3^). Indeed, the VEP performance of AlphaMissense as evaluated on a more recent release of ClinVar (Mar 2025) shows a strong dependence on the minor allele frequency (MAF, gnomAD v4^44^) of the included variants (Fig. 4d; Methods). VESM-3B, on the other hand, maintains the same performance independent of MAF, outperforming AlphaMissense in the clinically challenging task of distinguishing between rare benign and rare pathogenic mutations (for example, MAF <1 × 10^−3^). A similar conclusion can be drawn when explicitly removing all gnomAD v2 human variants with MAF > 1 × 10^−5^ that have been used for training AlphaMissense (Fig. 4e and Extended Data Fig. 5c). Interestingly, all VESM models (3B, 650M, 150M and 35M) are able to surpass the performance of AlphaMissense on this benchmark, substantially outperforming the corresponding base ESM models both in terms of AUC and other binary classification metrics (Fig. 4f and Extended Data Fig. 5d; Methods).

### Incorporating structure information further improves VESM on both clinical and DMS benchmarks

Having developed sequence-only PLMs with substantially improved performance compared to existing structure- and MSA-based models, we next investigated whether incorporating structural information could provide additional gains. In principle, the amino acid sequence of a protein encodes all the information necessary to determine its structure, suggesting that sequence-based models alone might suffice. However, both sequence and structure data are limited in practice, offering incomplete but potentially complementary views of the protein fitness landscape. Although joint sequence-structure pretraining could theoretically outperform sequence-only methods, our clinical VEP results (Fig. 4) suggest that existing models using this strategy (for example, SaProt, ProSST and ESM3) may underutilize sequence information when structure is readily available during training. More recently, structure-aware fine-tuning of sequence models was proposed as a promising approach to incorporate structure information in sequence-only PLMs, but the resulting model (ISM^45^ based on ESM2) underperformed on our ClinVar evaluations compared to SaProt (also based on ESM2).

**Fig. 5:**
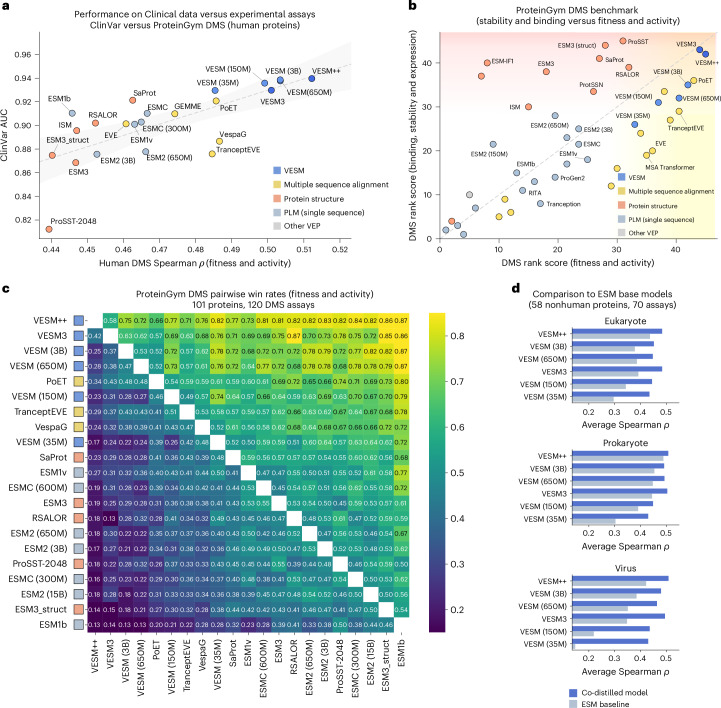
VESM achieves state-of-the-art VEP performance in both clinical and DMS benchmarks. **a**, A comparison of model performance on clinical VEP (ClinVar AUC, same dataset as in Fig. 4a) versus experimental VEP from human protein DMS assays (Spearman correlation, ProteinGym fitness and activity assays). Points denote individual models; the dashed gray line shows the fitted linear regression. Shaded bands (95% CIs) around the regression indicate uncertainty in the fit. The models are color-coded based on the additional sources of information they use. **b**, Performance on the ProteinGym DMS benchmark (rank score), with assays categorized by selection type: fitness/activity (*x* axis) versus binding, stability and expression (*y* axis). VESM3 and VESM++ achieve state-of-the-art performance across both assay types, surpassing existing structure- and MSA-based methods. **c**, Pairwise win rates between models across 120 DMS fitness and activity assays, quantifying the proportion of assays in which a given model outperforms another. **d**, A comparison of VESM models and ESM baselines across nonhuman DMS assays, grouped by taxonomic category (eukaryote, prokaryote and virus). Base models for individual VESM models: ESM2 (3B), ESM2 (650M), ESM2 (150M), ESM2 (35M) and ESM3_struct. For VESM++ the baseline is given by the ensemble of the corresponding base models ESM2 (3B) and ESM3_struct.

Here, in line with our proposed sequence-only training framework, we considered an alternative approach. Rather than using structural supervision to improve a sequence model, we instead use VESM to enhance the sequence representation within a structure-based model architecture. We selected ESM3 for its modular design, which cleanly separates sequence and structure components, and fine-tuned only the sequence-related parameters with the same sequence-based mean squared error (MSE) loss used within our co-distillation framework. This strategy allowed us to distill the benefits of VESM into a structurally informed model with minimal overhead, resulting in improved performance across both clinical and DMS VEP benchmarks (Fig. 5; Methods). We refer to the fine-tuned version of ESM3 as VESM3 and further combine it with the sequence-only VESM-3B to produce a structure-aware ensemble model, named VESM++.

To evaluate the models’ performance on human proteins, we curated a human DMS dataset from ProteinGym consisting of 50 assays measuring protein fitness and assessed the concordance between performance on ClinVar (AUC) and human DMS (Spearman correlation) benchmarks (Fig. 5a). Our results demonstrate that model improvements in clinical VEP translate to corresponding performance gains on human DMS assays, with a Pearson correlation of 0.78 between the two benchmarks. Notably, VESM3 outperformed its base model, ESM3, by a large margin on both clinical and DMS evaluations, achieving performance comparable to VESM-3B. The combined model, VESM++, further improved performance relative to individual VESM models. All individual VESM models, including VESM-35M, matched or surpassed the performance of all other MSA- and structure-based methods across both benchmarks.

We next expanded our DMS benchmark to include nonhuman proteins and categorized assays by primary readout: fitness/activity versus structure-dependent measures such as binding, stability and expression (Fig. 5b). This stratification highlights the distinct contributions of MSA and structural information to model performance; MSA-based models performed much better on fitness and activity assays, whereas structure-based models primarily improved performance on binding and stability-related tasks. Currently available PLMs that do not incorporate structure or MSA information (for example, ProGen2^46,47^, Tranception^48^, ESM2 or ESM-C) appeared to have reached a performance plateau on both assay types, independent of model size and architecture. By contrast, our sequence-only VESM models notably surpassed this barrier, matching the performance of MSA-based methods. Incorporating structure information further improved upon the sequence-only VESM models, with VESM3 and VESM++ simultaneously achieving state-of-the-art performance across both assay categories.

Pairwise evaluations across all ProteinGym fitness and activity DMS assays (Fig. 5c) further quantified the improved performance of VESM models, as measured by pairwise win rates (that is, the fraction of assays in which one model outperformed another; Methods). Our results showed that VESM++ and VESM3 achieve the highest overall win rates, outperforming both MSA- and structure-based models. This is also the case for sequence-only VESM models, except for the smaller (150M and 35M parameter) models, which still consistently outperformed all structure-based models. Relative to their respective base models, VESM3 surpassed ESM3_struct in 85% of assays, VESM-650M surpassed ESM2_650M in 78% and VESM-3B surpassed ESM2_3B in 79%.

**Extended Data Fig. 6:**
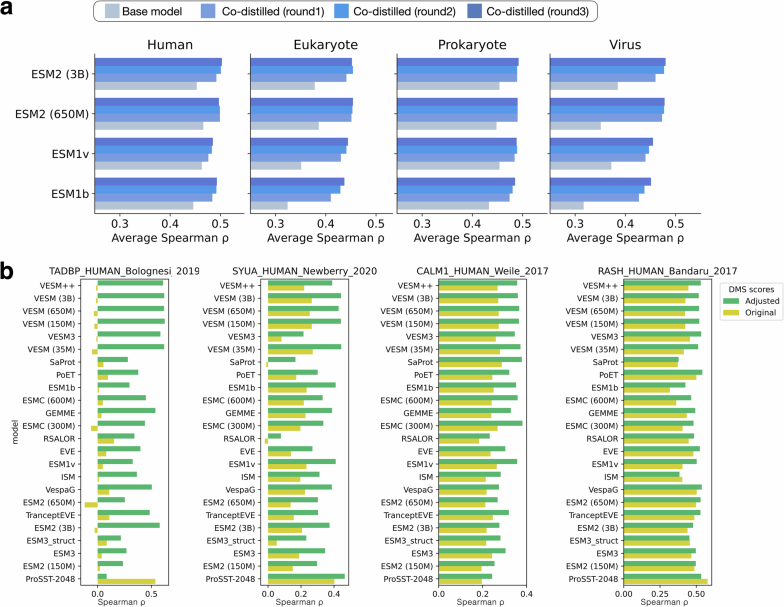
Additional analyses for the DMS benchmark. (**a**) Cross-taxon gains from co-distillation. Average Spearman correlation for base versus co-distilled models across rounds, showing disproportionally large improvements on viral assays despite round-1 training using only human sequences. (**b**) Performance improvements from adjusted DMS scores. Performance comparison across methods on four DMS organismal fitness and activity assays (TADBP, SYUA, CALM1, and RASH), where applying the score transformation x → abs(x - WT) led to the most substantial differences in performance across methods. Bars represent the Spearman correlation between model predictions and experimental DMS scores, using either the original data (yellow) or the adjusted data (green). The transformation accounts for assay-specific contexts in which higher-than-wild-type variant measurements are associated with deleterious effects.

Finally, examining model performance improvements across taxonomic categories revealed disproportionately larger gains for viral proteins relative to other nonhuman proteins (Fig. 5d) Similar gains were observed for ESM1b, ESM2 and ESM1v models during the first round of co-distillation (Extended Data Fig. 6). This is surprising, as these models were co-distilled exclusively on human protein sequences (Methods). Moreover, these improvements transferred effectively to VESM3 through VESM-based distillation, even though the corresponding ESM3 base model and its associated training data explicitly excluded all viral sequences before public release^35^.

### VESM can predict the severity of missense variant effects on continuous clinical phenotypes in the UK Biobank data

Although traditional VEP methods primarily focus on binary pathogenicity classification, extending these predictions toward quantitative, clinically relevant outcomes offers substantial potential for human genetics applications. To investigate whether VESM scores can quantify the severity of missense variants on continuous clinical traits, we analyzed genotype-phenotype summary statistics from the UK Biobank^49^ as provided by Genebass^50^, focusing specifically on genes associated with blood biochemistry biomarkers.

We downloaded summary statistics for all 332 significantly associated gene–phenotype pairs involving 186 genes and 27 distinct clinical phenotypes. Each gene was selected to have a minimum of 25 missense variants with Genebass estimated single-variant association effect sizes (*β* coefficients) and *P* values. We then examined the relationship between variant-level VESM scores and their observed clinical impact by performing linear regression of VESM predictions against the reported *β* coefficients, quantifying model accuracy at the gene–phenotype level using the Pearson correlation coefficient and its corresponding regression-derived *P* value (Methods).

**Fig. 6:**
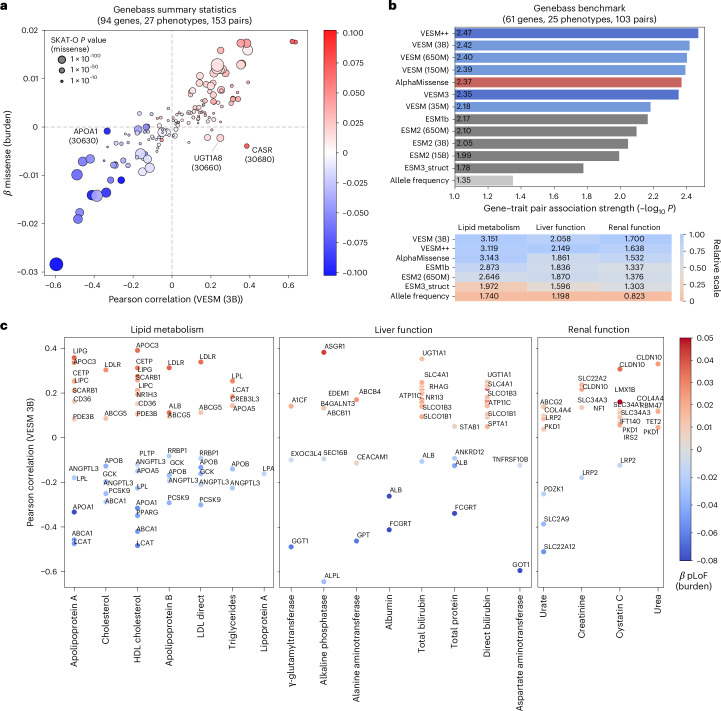
VESM models accurately quantify missense variant severity across continuous clinical phenotypes in UK Biobank. **a**, A correlation between variant-level VESM-3B predictions and single-variant association effect sizes (Genebass *β* coefficients) across 153 gene–phenotype pairs. The circle size represents missense SKAT-O significance (gene-level association *P* value from Genebass); color indicates gene-level pLoF effect size (burden test; Genebass). Prominent outliers are highlighted. **b**, Performance comparison of VESM models against AlphaMissense, ESM base models and an allele-frequency baseline across 103 strongly associated gene–phenotype pairs (Methods). Performance is summarized by average association strength (−log_10_ regression *P* value (top)) and stratified by phenotype category (bottom). Regression-derived *P* values were computed using Pearson’s product-moment correlation (two-sided test; scipy.stats.pearsonr); no adjustment for multiple comparisons was applied. **c**, A Pearson correlation between VESM-3B variant-level predictions and single-variant association effect sizes for gene–phenotype pairs with regression derived *P* value < 0.1 (two-sided test; as in **b**). Phenotypes are grouped by phenotype category (lipid metabolism, liver function and renal function), and the color denotes pLoF burden test effect size.

Remarkably, VESM-3B derived correlations strongly aligned with gene-level missense burden test effect sizes and SKAT-O^51^ *P* values across the majority of tested gene–phenotype pairs (Fig. 6a), underscoring the model’s capability in capturing not just pathogenicity but the magnitude and directionality of clinical impact. Upon closer examination of initial outliers, we observed that VESM-3B predictions corresponded more closely to burden test results derived from predicted loss-of-function (pLoF) variants rather than missense variants alone. These examples include well-established associations of APOA1 with apolipoprotein A levels, UGT1A8 with bilirubin metabolism and CASR with calcium levels. Importantly, the observed alignment between VESM correlations and pLoF-based effect sizes for these gene-trait pairs shows that VESM predictions can capture clinically relevant missense variant effects that traditional missense SKAT-O analyses may underestimate. We highlight that this regression approach, unlike SKAT-O, can be used to test for association between genes and phenotypes with only summary statistics.

**Extended Data Fig. 7:**
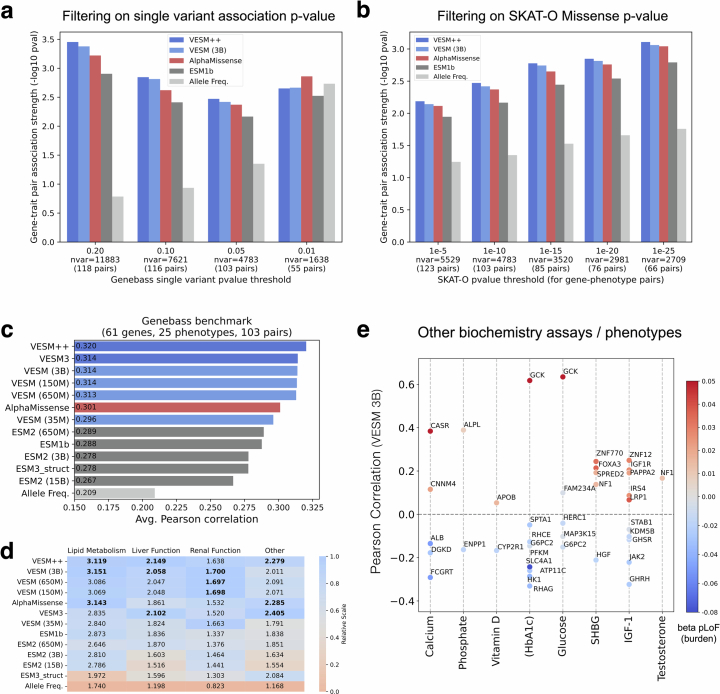
Genebass summary statistics. (**a**) Gene-phenotype association strength (-log10 p-value) for each model, assessed across varying single-variant p-value thresholds from Genebass summary statistics. (**b**) Association strength across different SKAT-O missense p-value thresholds used to define gene-phenotype pairs. VESM + + and VESM-3B consistently outperform alternative models across thresholds. (**c**) Average Pearson correlation between predicted variant scores and Genebass β coefficients across 103 high-confidence gene-phenotype pairs. (**d**) Average association strength (-log10 regression p-value; two-sided test; scipy.stats.pearsonr) of all VESM and baseline models across phenotype categories, including gene-phenotype pairs outside the three main categories shown in Fig. 6b. (**e**) Correlation between VESM scores and Genebass effect sizes for gene-phenotype pairs not included in the main phenotype categories (complementing Fig. 6c).

To compare VESM-3B with other models, we removed nonsignificantly associated missense variants (single-variant association *P* value >0.05) and further filtered the gene–phenotype pairs to include only those with strong associations (missense SKAT-O *P* < 1 × 10^−10^). This yielded 103 gene–phenotype pairs spanning 61 genes and 25 phenotypes. For each model, we computed the regression *P* value between predicted variant scores and Genebass effect sizes and summarized performance using the average −log_10_(*P* value) across all pairs to reflect the strength of variant-phenotype association captured by each model (Fig. 6b). VESM++ achieved the highest average association strength, followed closely by VESM-3B; both surpassed AlphaMissense and the ESM base models, as well as a simple allele-frequency predictor that was included as a baseline (log-MAF regression versus *β*). This relative ranking remained stable when varying the corresponding filtering thresholds and when the mean Pearson correlation was used instead of log *P* value as the performance metric (Extended Data Fig. 7a–c). Stratifying the analysis by phenotype category (lipid metabolism, liver and renal function) yielded similar results, demonstrating the robustness of VESM models across different biological contexts (Fig. 6b, bottom, and Extended Data Fig. 7d).

Finally, we assessed the predictive performance of VESM-3B across individual phenotypes by reporting the regression coefficients (Pearson correlation) between VESM scores and Genebass effect sizes for 169 gene–phenotype pairs that were identified by VESM as significant (*P* value <0.1; Fig. 6c and Extended Data Fig. 7e). VESM-3B captured the correct pLoF direction of effect in 167 of these pairs (98.8%) and the fitted regression coefficients aligned closely with the magnitude of the corresponding pLoF burden estimates (Pearson *ρ* = 0.883). Importantly, 54 of the 167 correctly oriented associations (32%) were not identified by the missense burden test, highlighting the additional sensitivity gained from modeling variant-level functional effects with VESM.

## Discussion

In this work, we explored the limits of sequence-only PLMs and developed an efficient approach to enhance their VEP accuracy without relying on additional structural or MSA information. Our co-distillation framework leverages a simple yet powerful principle that exploits the complementarity observed among models in the ESM family. During training, all participating models learn collectively by distilling predictions from the most confident model for each variant.

By selecting the model with the most confident prediction, or, equivalently, the minimum LLR for each variant, we effectively amplify distinct evolutionary signals captured during pretraining across the entire model family. Our theoretical analysis showed that compared to the standard practice of ensemble averaging, minimum-LLR aggregation should be preferred when predictions for true positives (pathogenic variants) vary more across models compared to those for true negatives (benign variants). Under this variance asymmetry, which we empirically observe in the ESM family, minimum-LLR aggregation can increase class separation more than averaging reduces within-class variance, yielding better classification performance.

We then demonstrated that a single round of maximum-confidence co-distillation substantially improves the performance of all ESM models across both clinical and experimental VEP benchmarks, with larger models individually matching or surpassing the performance of the aggregated signals used to train them (ESMIN). Smaller ESM models also benefitted from co-distillation, showing disproportionately large gains for their size (for example, ESM2‑8 M AUC rising from ~0.65 to ~0.88). Multiple ablation studies confirm that these performance gains are robust to (1) which base models are used, (2) the size of the training dataset and (3) the level of sequence homology between training and test sets. For example, co-distillation with only 1% of the data (~200 sequences with less than 30% identity to the test set), already allows ESM2-35M to achieve >94% of its full performance on both benchmarks.

We further found that additional rounds of co-distillation continue to improve performance, eventually allowing a single model to match the co-distilled ensemble. As predicted by our analysis, once class separation is high and class-conditional variances become more symmetric, switching to simple averaging in later rounds yields small but consistent gains compared to using minimum-LLR (maximum-confidence) aggregation throughout. Crucially, ablations that replace the first-round maximum-confidence step with averaging at every round performed substantially worse, indicating that the initial minimum-LLR round is necessary to achieve the downstream improvements on both clinical and experimental VEP benchmarks. The output of this iterative process is VESM-3B, which effectively compresses the collective knowledge of the ESM family into a single PLM. Further distillation from VESM-3B produces smaller VESM models (650M, 150M and 35M parameters) that retain most of the performance at a fraction of the computational cost, enabling high-throughput inference and practical use in resource-limited settings.

To provide a comprehensive evaluation of the resulting VESM models we compared their performance against state-of-the-art PLMs and VEP methods that incorporate additional information from MSA, structural data or population-level priors. Remarkably, despite being trained exclusively on unaligned sequence data, VESM models outperform more complex hybrid methods, including PoET, TranceptEVE and SaProt. Notably, VESM-3B also surpasses the performance of AlphaMissense, a recent state-of-the-art VEP model trained on structural, evolutionary, and population-level information.

Importantly, our direct comparison with AlphaMissense highlights the robustness of VESM in predicting variant effects across the entire spectrum of allele frequencies. Unlike models that incorporate population-based features and risk circularity in clinical annotation, VESM-3B maintains consistent performance independent of allele frequency information, enabling more accurate discrimination between rare pathogenic and rare benign variants. This capability is critical for clinical genetics and exome sequencing studies, where actionable insights often rely on interpreting ultrarare missense mutations with limited prior annotation.

Although VESM achieves exceptional performance with sequence information alone, we further demonstrated that structural information can be incorporated in a lightweight, modular fashion without requiring complete model retraining. By distilling VESM-3B onto the sequence backbone of ESM3, we created VESM3, a hybrid model that inherits ESM3’s structural inductive biases while benefiting from the robust evolutionary representations learned through VESM. This results in a single model that simultaneously achieves state-of-the-art performance across both structure-dependent assays (protein stability, binding and expression) and fitness/activity assays, effectively overcoming the performance trade-offs observed in existing PLMs.

Our findings reveal a strong correspondence between improved performance on clinical variant classification and increased predictive accuracy on human DMS assays. The correlation between these benchmarks suggests that the evolutionary signals captured by our models effectively translate across both clinical and experimental contexts. Notably, the performance gains extended to nonhuman proteins and across broad taxonomic groups, even when co-distillation was limited only to human sequences. This cross-domain generalization further validates VESM’s utility as a foundational VEP model.

Finally, we demonstrate that VESM predictions quantitatively reflect the severity of variant effects on continuous clinical phenotypes, expanding their utility in human genetics applications. Variant-level VESM scores correlate strongly with effect sizes from rare variant association studies and recapitulate gene-level burden signals from SKAT-O tests. Importantly, VESM predictions align more closely with gene-level pLoF burden signals, suggesting that our models capture functionally relevant aspects of protein variation that extend beyond binary pathogenicity classification.

## Methods

### Enriching for evolutionary signals within the ESM family of models

#### Scoring variant effects

ESM models are pretrained with the masked language modeling objective to predict probabilities of amino acids being at particular positions in protein sequence. Based on this property, we follow the previous work^21^ to score the effect of a mutated sequence ${x}^{\mathrm{mt}}$ by comparing it with the corresponding WT sequence ${x}^{\mathrm{wt}}$ using LLR, as follows:

$$\mathrm{LLR}({x}^{\mathrm{mt}}|{x}^{\mathrm{wt}})=\mathop{\sum }\limits_{t\in T}\log p({x}_{t}={{x}_{t}^{\mathrm{mt}}}|{x}_{\backslash t})-\log p({x}_{t}={{x}_{t}^{\mathrm{wt}}}|{x}_{\backslash t}),$$

where *T* denotes the set of mutated positions, $x^{\mathrm{wt}}_t$ and $x^{\mathrm{mt}}_t$ denote the wild-type and mutant amino acids at position *t*, and $p({x}_{t}=\alpha|{x}_{\backslash t})$ denotes the probability of amino acid $\alpha$ predicted by the model at position *t* conditioned on the rest of the sequence. Intuitively, in terms of variant effect, the LLR score represents the benign degree of a mutation compared to the reference sequence. Higher scores indicate more benign and vice versa. Following the WT marginal probability scheme^21^ for inference efficiency, we perform a single forward pass on the WT sequence to score the probabilities and calculate the score for all mutations.

#### Evolutionary signal enrichment and the ESMIN dataset

Given a set of pretrained ESM models, for each mutation, we calculate LLR scores for all models and take the minimum LLR score (that is, most pathogenic or damaging prediction across all models) to create a pseudo-LLR score for the mutation. This approach selects the model with the maximum confidence in the WT residue relative to a given substitution for that position, effectively prioritizing models that have identified the corresponding residue as part of a mutationally sensitive, evolutionarily conserved motif. Following the above process, we create the ESMIN dataset with 11 ESM models (ESM2: 8M, 35M, 150M, 650M and 3B; ESM1b; and five ESM1v models) (Supplementary Table 1), using 20,284 manually reviewed human protein sequences from UniProt (February 2022)^52^. For proteins longer than 1,022 amino acids (maximum context window of ESM1b and ESM1v models), we follow a sliding window approach^6^ to segment them into 1,022-residue windows with an overlap of 511 residues. For each protein, we compute scores for all possible $19\times L$ missense variants (where *L* is the protein length), resulting in ~197 million variants in total, across 2.11 × 10^4^k segments. We further filter out noninformative protein segments (that is, segments whose ESMIN scores are all similar in a range close to zero), by first computing the minimum ESMIN score across all variant positions (except the first) for all segments and then filtering out segments whose minimum ESMIN score is higher than the 95th percentile of the distribution. This results in a total of 19,600 segments across 18,683 proteins with a total of 191.3 million unique variant scores. The resulting ESMIN scores range from −31.2 to 7.02, with the score of the WT variants being 0.

### Maximum confidence co-distillation and training of VESM models

We develop a co-distillation framework to improve all individual sequence-only models within the ESM family (Supplementary Table 1). Simplifying notation from the previous section, we let $V$ denote the set of all possible mutations in the human proteome and $F({v|G})$ be the corresponding log likelihood ratio assigned to the variant $v$ in $V$ by model $G$. The main iteration for co-distillation of $n$ models ${G}_{1},\ldots ,{G}_{n}$ using MSE as the loss function, is given by

For variant $v$ in $V$ and model ${G}_{i}$, i = 1, .‥, n:

$${\mathrm{Loss}}_{i}$$ = MSE($$F({v|}{G}_{i})$$, $$\mathop{\min \,}\limits_{s}F({v|}{G}_{s})$$)

backpropagate($${\mathrm{loss}}_{i}$$, model $${G}_{i}$$).

To avoid training $n$ models in parallel and to bypass the need to recompute $\mathop{\min \,}\limits_{s}F({v|}{G}_{s})$ scores in each iteration, we consider a simplified approach that precomputes and fixes $\mathop{\min \,}\limits_{s}F({v|}{G}_{s})\triangleq$ ESMIN($v$) for all iterations. In this case, co-distillation is performed by the distillation of the precomputed ESMIN scores to each individual model that contributed to its computation, that is, by setting in the above, ${\mathrm{loss}}_{i}$ = MSE($F({v|}{G}_{i})$, ESMIN($v$)). Finally, for better training convergence and to maintain co-distilled model scores in a similar range with respect to their pretrained versions, we shift the ESMIN target scores for each model to match the empirical mean of $F({v|}{G}_{i})$ across all variants $v$ in $V$. These shifts are calculated once using the LLR scores from the base ESM models and are kept constant during training.

#### Parameter-efficient training

We freeze the low-level layers of the pretrained ESM models and fine-tune only: (1) the last hidden layer and (2) the language model head. The trainable parameters account for a small fraction of the total number of parameters, for example, approximately 3.3% and 2.8% of the total parameters for ESM2 (650M) and ESM2 (3B) models respectively. All models were trained with exactly the same settings for five epochs. Following standard practice for fine-tuning pretrained encoders^53,54^, we scale the learning rate based on training duration and use AdamW with a rate of 4 × 10^−4^, which is obtained by adjusting a standard 1 × 10^−3^ rate to a five-epoch schedule. AdamW *β* are set to (0.9, 0.999) and weight decay is 0.01, and we apply a cosine scheduler with a 5% warmup. We randomly split the proteins using a small set of 200 segments for validation and the rest (19,434 segments) for training.

#### Computing resources

All experiments were conducted on a single NVIDIA H100-SXM GPU (81.5-GB VRAM) per run. The workstation has 4xH100-SXM, but no model/data parallelism used for training. For all training runs we used: Python (3.12.3), PyTorch (2.3.0), CUDA (12.1), cuDNN (8.9.7) and mixed precision (FP16). All other packages used are included in the ‘environment.yml’ file located in our github repository (https://github.com/ntranoslab/vesm). Training of the largest ESM-3B model on our system with the above settings (last layer + head; 85.4 million trainable parameters) took ~18 h with peak VRAM measured at ~77 GB. For comparison training of the 35M-parameter model took only 35 min. See Supplementary Table 2 for more details.

### Assessing the generalization capabilities of the co-distilled models

To evaluate the generalization capability of our co-distilled models, we conduct an ablation study by varying the percentage of protein sequences used for training. First, we exclude from the training pool all human proteins sharing more than 30% sequence similarity with any protein in our evaluation benchmarks (2,400 proteins in Balanced ClinVar and 101 proteins in DMS organismal fitness and activity assays). To do this, we merged the training and benchmark sequences and clustered them with MMseqs2^55,56^ (easy cluster; release 13-45111) using --min-seq-id 0.30, --cov-mode 0 (coverage relative to the longer sequence), -c 0.8 (≥80% aligned length) and --cluster-mode 1 (connected-component). We then removed any training sequence that coclustered with a benchmark member. This filtering step excludes 5,279 sequences from the full training set of 18,683 human proteins. From the remaining 14,355 proteins, we create nested subsets of decreasing size (50%, 10%, 5% and 1% of all sequences) to assess model performance with limited training data. Subset sampling was repeated five times with independent random seeds. For each subset, we fine-tuned three ESM2 models (8M, 35M and 150M) using a 90%/10% training/validation split and following the same training settings described above.

### Fine-tuning on downstream tasks using VESM model embeddings

To assess the effect of our co-distillation framework on the protein representation capabilities of ESM models, we evaluate the performance of the embeddings from both the base and co-distilled versions of ESM2-650M (VESM2) on nine downstream tasks across four categories: (1) function prediction: thermostability prediction task from FLIP^57^ that predicts the thermostability level of proteins, metal ion binding prediction^58^, (2) localization prediction: subcellular localization^59^ with two location categories (DeepLoc-2, binary classification) and ten location categories (DeepLoc-10, multiclass classification), (3) protein–protein interaction prediction: human protein–protein interaction (HumanPPI) prediction^59,60^, (4) mutational effect prediction: adeno-associated virus fitness^57^, β-lactamase activity^59^, fluorescence prediction and protein stability tasks from TAPE^61^. We follow the same train/valid/test splits provided by SaProt^14^.

We freeze the embeddings from both models and train a (head) neural network built on the top of ESM’s embedding. For Thermostability, HumanPPI, MetalIonBinding, DeepLoc-2 and DeepLoc-10, we use the ESMClassification module and the same fine-tuning framework as in SaProt^14^. For the rest of the tasks, we design a similar two-layer network on top of the ESM’s embedding that is a sequence of a linear layer (from embedding size to embedding size) and a LayerNorm layer, followed by a GELU activation and a linear projection layer. We train the network using the AdamW optimizer with *β* = (0.9, 0.98) and a cosine linear scheduler with learning rate of 1 × 10^−4^. We use MSE loss for regression tasks and cross entropy loss for classification tasks. For all tasks we repeat the training three times with different random seeds and report the mean and std of each model’s performance.

### Multiple rounds of co-distillation

We selected the top four co-distilled models from the first round (ESM2-3B, ESM1b, ESM2-650M and ESM1v5) and performed two additional rounds of co-distillation (rounds 2 and 3; Fig. 3b). The co-distillation process was kept the same as round 1, with the only difference being that per-variant LLR scores across the participating models were aggregated by averaging instead of taking the minimum. In round 2 we co-distilled these four models on the same set of human proteins used in round 1. For the third round, to increase learning capacity we repeated averaging co-distillation using a dataset exclusively composed of nonhuman proteins (see below). For all rounds, training continued from each model’s previous-round checkpoint.

#### Training setting

To increase model capacity while retaining efficiency, we fine-tune the head and the last three embedding layers. This raises the number of trainable parameters close to 8% for ESM2 (3B). We lowered the learning rate to 1 × 10^−4^ to continue training from the previous round. Weight decay remains 0.01 but is applied only to primary weight matrices (excluding biases and normalization parameters), following best practices to mitigate forgetting and maintain training stability^62^. The same cosine scheduler with 5% warmup is used.

#### Nonhuman protein sequences

To generate the nonhuman dataset we started from a UniProtKB reviewed export (downloaded 20 February 2025) and first compiled the set of human protein-family names by parsing the ‘protein families’ field of *Homo sapiens* records. We then retained only nonhuman entries that (1) had an InterPro accession, (2) were less than 1,022 amino acids long and (3) whose own family label was not in the human set (entries lacking a family label were not excluded by this step). To remove redundancy, remaining sequences were ranked by an evidence score derived from UniProt the ‘protein existence’ field (where 1 is the protein level, 2 is the transcript level and 3 is the homology), with a +0.5 penalty if the family label was missing, and the top-ranked sequence per InterPro accession was kept. This process yielded a dataset of 23,803 nonhuman proteins that was used both in the final round of co-distillation, as well as in all downstream knowledge distillation steps (see below).

#### Multiround ablation study

We ablated the aggregation operator used in each round, denoting a schedule as agg_1_–agg_2_–agg_3_ (for example, min–avg–avg means minimum aggregation in round 1, average in rounds 2 and 3). We evaluated three aggregation combinations: (1) avg–avg–avg, (2) min–min–min and (3) min–avg–avg, across all three rounds, reporting intermediate results where, min–avg for example denotes the corresponding result of the second round (Extended Data Fig. 4). We note that to follow the same setting we used for our main results, round 1 co-distillation was performed by aggregating LLRs across all 11 models, not only the four used for the subsequent rounds. Across both benchmarks, and all four models evaluated, using maximum confidence in round 1 was essential for achieving the strongest downstream performance. Averaging across multiple rounds was not able to further improve performance. After using minimum aggregation in round 1, the choice between averaging and minimum had a smaller effect, with averaging in rounds 2 and 3 achieving the best overall results (Extended Data Fig. 4b).

### Distilling VESM-3B into 650M-, 150M- and 35M-parameter models

To broaden accessibility across compute budgets, we distilled the converged model from round 3 (VESM-3B) into the base (non-co-distilled) ESM2 backbones at 650M, 150M and 35M parameters. First, VESM-3B was used to compute target LLR scores on the union of the human and nonhuman protein sets described above, and then each ESM2 model was trained on these target LLRs with an MSE loss, as described in our co-distillation framework. For this knowledge distillation step, we fine-tuned the full models for ESM2-35M and ESM2-150M, and for the larger ESM2-650M, we fine-tuned the output head and the last five embedding layers (~100 million parameters). We used the same optimization setup as in the multiround training: AdamW with *β* (0.9, 0.999) and a cosine learning rate scheduler with 5% warmup. The learning rates were set as follows: 1 × 10^−3^ for ESM2-35M, 1 × 10^−3^ for ESM2-150M and 1 × 10^−4^ for ESM2-650M. The resulting models are referred to as VESM-35M, VESM-150M and VESM-650M.

### Distilling VESM-3B into ESM3

Having established our co-distillation framework using sequence-only models from the ESM family, we next extend this approach to the sequence component of a multimodal protein model. Specifically, we apply our framework to the ESM3^35^ model (esm3-sm-open-v1; 1.4B parameters; https://huggingface.co/EvolutionaryScale), which integrates multiple modalities including sequence, structure, solvent accessibility, secondary structure (SS8) and functional annotations. Our objective is to fine-tune only the sequence-related modules of ESM3, while keeping the remaining components fixed at their pretrained weights.

For training, we construct input-output pairs using protein sequences and VESM-derived LLRs, to distill the VESM (3B) model into the sequence module of ESM3. To increase training diversity, we use both sets of human and nonhuman protein sequences described above. We note that no 3D structure or any other modality other than sequence is used as input during training. Since ESM3 uses the same head for structure conditioned and sequence only inference, we keep the head parameters frozen during training and fine-tune only the last five embedding layers of the model. We use the same optimization setup as above (knowledge distillation, ESM2 models) with learning rate 5 × 10^−5^.

During inference, we enable both sequence and 3D structure inputs to the fine-tuned model. The structural input is processed using ESM3’s default configuration (pretrained weights), resulting in the VESM3 model described in the Main. We note that the use of 3D structure as input to VESM3 during inference is optional and that the fine-tuned model retains the capacity to accept all eight input modalities supported by ESM3. In this work, we only evaluated performance of VESM3 using 3D structure as input. To obtain VESM++ scores, we average the LLRs from VESM3 and VESM-3B, that is, LLR(VESM++) = (LLR(VESM3) + LLR(VESM-3B))/2.

### Inference runtime

All VESM models developed in this work inherit the computational efficiency of their corresponding base models within the ESM family. To provide guidance with respect to compute resources required for using our models in practice, we conducted a set of runtime experiments reporting peak VRAM (GB), throughput (seq s^−1^) and latency (ms) across three different sequence lengths (short, 254 amino acids; medium, 510 amino acids; and long, 1,022 amino acids). Supplementary Table 3 provides the full results for the 35M-, 650M- and 3B-parameter models (sequence-only VESM), as well as VESM3 with sequence + structure input. We also included ESM2-15B for reference. Batch size was set to 16 for all experiments and for each metric we reported the average over steady-state windows after warmup, with standard deviation across two runs.

For example, we can use the above results to compare runtime estimates for calculating the LLRs of all possible single missense mutations in a set of 2.5 × 10^4^ protein sequences, where each one is ~1,000-amino-acids long (that is, ~450 million variant effect scores in total). For this reference dataset, VESM-3B would need ~1 h of inference time, while consuming less than 16 GB of VRAM. VESM-650M would need ~20 min and the smallest sequence-only VESM model (35 million parameters) would complete this task in around 3 min. For comparison, VESM3 would also need ~40 min but would require a much larger VRAM memory footprint measured at ~46 GB. We note that all of our experiments were performed on our local workstation (4xH100-SXM GPU) so while absolute wall-clock time estimates may vary when performed on other systems/configurations, the relative performance across models and the peak VRAM requirements will be more directly comparable with the estimates provided above.

### Datasets

#### ProteinGym ClinVar

The dataset consists of 62,656 missense variants covering 2,525 human genes. To avoid data circularity, we excluded supervised methods and meta-predictors that have been trained on clinical labels (ClinPred, VEST4, REVEL, MetaRNN, BayesDel and MutationTaster) and methods with more than 10,000 missing values. We then kept all variants for which all methods have available predictions. This resulted in a dataset with 52,637 variants (26,897 pathogenic and 25,740 benign) across 2,227 genes that we used for our evaluation. We also generated a subset of this dataset (referred to as Balanced ClinVar), by subsampling majority class labels to match the number of pathogenic and benign variants for each protein. This balanced version of ClinVar includes a total of 27,468 variants across 2,400 proteins.

#### Full ClinVar dataset download

To compare with AlphaMissense we downloaded the latest release of ClinVar (March 2025 version) and computed MAFs for all variants using gnomAD v4. The ClinVar dataset was filtered to include all hg38 SNVs with predicted effects on RefSeq NM ids. To map between hg38 coordinates, RefSeq and UniProt accessions, we used the latest RefSeq annotation release (GCF_000001405.40-RS_2024_08) and the UniProt ID mapping server (available at uniprot.org/id-mapping). For quality control we kept variants annotated with at least 1 ‘Gold Star’, resulting in a dataset of 151,600 missense variants. For these variants we added MAF computed from gnomAD_v4 using the AF column, as MAF = min(AF,1-AF). For variants not included in gnomAD v4, we set MAF = 0. We also computed MAF from gnomAD v2 (v2.1.1, liftover_grch38) and annotated variants that have gnomAD v2 MAF >1 × 10^−5^ as ‘used for training AlphaMissense’. Finally, we mapped precomputed AlphaMissense scores using hg38 genome coordinates from AlphaMissense_hg38.tsv.gz (downloaded from https://www.doi.org/10.5281/zenodo.org/8208688). After filtering out missing values, we obtained a dataset of 142,951 variants (46,247 pathogenic and 96,704 benign) across 2,685 genes.

#### ProteinGym DMS

The DMS benchmark consists of 217 DMS assays covering 696,311 single variants. The assays are categorized by taxon (96 human, 40 eukaryote, 50 prokaryote and 31 virus) and coarse selection type (77 organismal fitness, 43 activity, 66 stability, 18 expression and 13 binding assays). For our evaluations we further combine them into structure related assays (stability, expression and binding, 97 total) and function related assays (fitness and activity, 120 total). We used the provided experimental scores for all DMS assays except for fitness and activity measurements of CALM1, TPK1, UBC9, RASH, TADBP, SYUA and SRC. For these assays we applied a transformation of the form *x* → abs(*x* − WT), where WT represents the WT measurement (either 0 or 1, depending on the assay). This adjustment reflects the fact that, for these assays, variants scoring higher than the WT are typically associated with deleterious effects, as discussed in the original studies^63^. This preprocessing step improved performance across all evaluated methods, with the exception of the structure-based model ProSST^19^, whose performance on TADBP dropped substantially, from 0.54 to 0.08 (Extended Data Fig. 6b). Although we were unable to pinpoint the cause of this discrepancy, it is unlikely to be related to structural features, as the regions assayed in TADBP are intrinsically disordered^64^.

#### Protein structure data for evaluation

For ProteinGym DMS, we used the 3D structures provided by the benchmark. For ProteinGym ClinVar, we used 3D structure provided by AlphaFold Protein Structure Database (https://alphafold.ebi.ac.uk) for 2,300 sequence-matched proteins and generated 3D structure for the remaining proteins using the AlphaFold2 script from ColabFold^65^ (https://github.com/sokrypton/ColabFold).

### Comparisons with state-of-the-art PLMs and VEP methods

We compared VESM models with 25 models on ClinVar and 39 models on the DMS benchmark. These baselines are the latest or widely used PLMs and VEP methods, including PLMs trained on unaligned sequences (for example, ESM, ProGen2 and Tranception), structure-based models (SaProt^14^, ESM3^35^, ProSST^19^ and ISM^45^), MSA-based models (PoET^20^, EVE^36^, TranceptEVE^18^, GEMME^37^, VespaG^38^ and RSALOR^61^) and other VEP methods that use homology-based (for example, SIFT^39^ and PROVEAN^40^) or population-based (for example, PrimateAI^13^ and CADD^42^) approaches. See Supplementary Table 4 for a detailed description.

For the ProteinGym benchmark, we obtained precomputed scores from ProteinGym for all methods except RSALOR^61^, ProSST, SaProt, ESM3, ISM, VespaG and ESMC. Among these models, ISM scores are currently not reported in the benchmark. Other models do not have scores for the ClinVar benchmark. Furthermore, we wanted to ensure that all structure-based models use the same set of 3D structures for evaluation. Therefore, we compute variant effect scores for all of the above models, following the inference scripts provided in the corresponding repositories. For the comparison with AlphaMissense^7^, we used the precomputed scores provided by the authors (https://zenodo.org/records/10813168).

### Performance evaluation on ClinVar

We evaluated performance on ClinVar using the area under the receiver operating characteristic curve (AUC) as our primary metric. We reported both global AUC (calculated across all variants) and average per-gene AUC (calculated separately for each gene with at least ten annotated variants). We bootstrapped the global AUC calculation in Fig. 4a, by randomly sampling 50 label-balanced sets of 6,000 variants (3,000 benign and 3,000 pathogenic). The plot shows the estimated mean and standard deviation.

#### Prediction accuracy versus variant annotation

For this analysis, the scores of each method were first calibrated by performing logistic regression on a small set of ClinVar variants (*n* = 1,000), randomly sampled to contain an equal number of benign and pathogenic labels. This step ensures that all the scores are comparable between 0 and 1, with 0.5 being the classification threshold. Then we varied the classification threshold symmetrically to exclude variants that each method is most uncertain about (with scores around 0.5). For each threshold, we computed the prediction accuracy, defined as the average accuracy of correctly annotating both benign and pathogenic variants, as well as the total number of variants that each method was able to annotate (that is, the ones that were not excluded by the threshold). Overall, this process was repeated ten times by randomly sampling the calibration set, and the two quantities (± 2 standard deviations around the mean) were plotted against each other in the figure.

#### Comparison with AlphaMissense/MAF filtering

We compared VESM models with AlphaMissense across different MAF thresholds (1 × 10^−5^, 1 × 10^−4^, 1 × 10^−3^, 1 × 10^−2^ and 1 × 10^−1^) to assess the impact of population frequency on model performance. For each threshold, we filtered the ClinVar dataset (Feb 2025 release) to include only variants below the specified MAF value and calculated the AUC for each model, by randomly sampling 100 label-balanced sets of 20,000 variants (10,000 benign and 10,000 pathogenic) from genes that contain both labels. Even at the lowest MAF threshold (1 × 10^−5^) the filtered ClinVar dataset remained well balanced with a total of 18,145 benign and 40,046 pathogenic variants across 2,559 genes. We also performed a specific comparison on the subset of ClinVar variants not used in AlphaMissense training, by removing gnomAD v2 variants with MAF > 1 × 10^−5^. This resulted in a set of 31,967 pathogenic and 15,392 benign variants across 1,510 genes. We emphasize that this filtering step is based on MAF calculated based on gnomAD v2 and does not exclude 4,818 variants that are reported in gnomAD v4 with MAF >1 × 10^−5^. As above the mean AUC and standard deviation was calculated over 100 bootstraps but with 10,000 (instead of 20,000) variants sampled in each iteration to account for the smaller size of the filtered dataset. On this dataset, we also computed binary classification metrics by first calibrating predictions for all models as in Fig. 4c (logistic regression, *n* = 1,000 variants) and using 0.5 as the classification threshold. The reported metrics are estimated by sampling the calibration set 50 times.

### Performance evaluation on DMS data

We evaluated model performance on the ProteinGym DMS benchmark using the weighted average of Spearman correlations between model predictions and experimental measurements, following the scripts and scoring guidelines outlined in the ProteinGym github repository^17^ (https://github.com/OATML-Markslab/ProteinGym). We compared all VESM models (3B, 650M, 150M, 35M, VESM3 and VESM++) against 39 other methods (see ‘Comparisons’ in Supplementary Table 4) across four datasets: (1) human DMS assays measuring fitness and activity (Fig. 5a), (2) all DMS assays measuring fitness and activity (Fig. 5b,c), (3) all DMS assays measuring binding, stability and expression (Fig. 5b) and (4) nonhuman DMS assays measuring fitness and activity (Fig. 5d).

To assess consistency between human DMS and ClinVar benchmarks, we calculated the correlation between model performances on the two datasets, considering only methods evaluated across both (Fig. 4a, Fig. 5b). In addition to average performance, we computed a rank score by sorting methods in ascending order from 1 to 43 based on their weighted average Spearman correlations (Fig. 5b). We also assessed pairwise win rates^66^ between models (Fig. 5c). For each pair of models, we computed the proportion of assays where the first model achieved a higher Spearman correlation than the second. Formally, the win rate of model ${m}_{1}$ over ${m}_{2}$, is defined as $\mathrm{win}\,\mathrm{rate}({m}_{1},{m}_{2})=\frac{1}{N}{\sum }_{i=1}^{N}1\{{S}_{i}({m}_{1}) > {S}_{i}({m}_{2})\}$, where ${S}_{i}(m)$ denotes the Spearman correlation achieved by model $m$ on assay *i*, $N$ is the total number of assays and $1\{\cdot \}$ is the indicator function. We note that although win rates were used in previous work^66^ to compare between models with nonoverlapping sets of VEP, here we compare models on exactly the same assays and variants.

### UK biobank summary statistics and the Genebass clinical phenotyping benchmark

#### Genebass summary statistics

We downloaded summary statistics (gene-level effect size and association *P* value) for the top pLoF gene burden associations with blood biochemistry biomarkers (pLoF SKAT-O *P* value <1 × 10^−6^) from the Genebass web portal (https://app.genebass.org/), including only genes with at least 25 missense variants. This resulted in a dataset of 332 gene–phenotype pairs across 186 genes and 27 biomarkers. For each gene–phenotype pair, we then downloaded the single-variant association summary statistics (174,073 effect sizes and *P* values across 80,245 distinct missense variants). Finally, we annotated the corresponding gene-level effect sizes and *P* values for 153 out of 332 gene–phenotype pairs that were identified as significant by Missense SKAT-O (*P* value <1 × 10^−6^).

#### Variant-level VESM score regression

For each gene–phenotype pair we performed a linear regression of the variant-level VESM predictions against the Genebass-derived single-variant effect sizes (*β* coefficients) and reported the standardized slope (Pearson correlation) as a proxy of the gene level effect size estimate and the corresponding VESM regression derived *P* value.

#### Clinical phenotyping benchmark

To create a benchmark based on Genebass summary statistics we wanted to ensure that the included gene–phenotype pairs contained detectable signals using missense variants alone. We therefore further filtered the dataset to include only (1) gene–phenotype pairs that were identified as significantly associated by Missense SKAT-O (*P* value <1 × 10^−10^) and (2) missense variants that were individually associated with the phenotype (single-variant association *P* value <0.05). This resulted in a dataset of 103 gene–phenotype pairs across 61 genes and 25 phenotypes. We evaluated methods on this dataset by applying the same linear regression approach described above to predictions derived from VESM (3B, 650M, 150M and 35M), VESM3 and VESM++, AlphaMissense, ESM1b, ESM2 (650M) and the log_10_-transformed MAF of the variants (Fig. 6b). To ensure robustness of the benchmarking results we also evaluated the above methods on datasets derived using different choices for the filtering thresholds (Extended Data Fig. 7a-b).

### License

We note that the predictions and weights of the VESM3 and VESM++ models are derived from fine-tuning the ESM3-small-open model developed by EvolutionaryScale which is available under a noncommercial license agreement (see https://github.com/evolutionaryscale/esm for detailed license options for these models).

### Reporting summary

Further information on research design is available in the Nature Portfolio Reporting Summary linked to this article.

## Online content

Any methods, additional references, Nature Portfolio reporting summaries, source data, extended data, supplementary information, acknowledgements, peer review information; details of author contributions and competing interests; and statements of data and code availability are available at 10.1038/s41592-026-03050-9.

## Supplementary information

Supplementary Information Supplementary note and Tables 1–4.

Reporting Summary

## Data Availability

Proteome-wide VESM predictions: precomputed VEP scores for VESM-3B, VESM3 and VESM++ are available via Hugging Face at https://huggingface.co/datasets/ntranoslab/vesm_scores. Specifically, we provide predictions for (1) all 71 million human protein coding SNPs based on hg38 and hg19 genome coordinates and (2) all possible 216 million missense mutations in the human proteome (all 20,421 human sequences from UniProt). We further make VESM predictions available in an interactive web portal via Hugging Face at https://huggingface.co/spaces/ntranoslab/vesm-variants.

## Code availability

Trained weights for all VESM models developed in this work (VESM-3B, -650M, -150M, -35M, and VESM3) are available via Hugging Face at https://huggingface.co/ntranoslab/vesm and are also deposited via Zenodo at 10.5281/zenodo.18777964 (ref. ^67^). The source code, scripts and data to reproduce all figures are available via GitHub at https://github.com/ntranoslab/vesm and https://github.com/ntranoslab/DJZN25 and are also deposited via Zenodo at 10.5281/zenodo.18393319, 10.5281/zenodo.18394714 (refs. ^68,69^) and via figshare at 10.6084/m9.figshare.31162471 (ref. ^70^). We also provide a Google Colab notebook that can be used to download the models and generate VEP scores for any protein sequence (https://colab.research.google.com/github/ntranoslab/vesm/blob/main/notebooks/VESM_Getting_Started.ipynb).
