# Supplementary material for: Compressing the collective knowledge of ESM into a single protein language model
Source: Nat Methods. 2026 Mar 30;23(4):772–84. doi: 10.1038/s41592-026-03050-9 (PMC13076216; doi:10.1038/s41592-026-03050-9)
Supplement: Supplementary file 1 — Supplementary note and Tables 1–4. [file 41592_2026_3050_MOESM1_ESM.pdf]

nature methods

Supplementary information https://doi.org/10.1038/s41592-026-03050-9

Compressing the collective knowledge of ESM into a single protein language model

In the format provided by the authors and unedited

Table of Contents

Supplementary Note	2
Supplementary Tables 1-4	10

Supplementary Note:

Mean vs Max Aggregation in Binary Classification Ensembles

Accompanying the manuscript: T. Dinh et al., "Compressing the collective knowledge of ESM into a single protein language model"

---

1 Problem Setup

We analyze binary classification ensembles with n base learners, where each learner $i \in \{1, \ldots, n\}$ produces a real-valued score S_i. Higher scores indicate stronger evidence for the positive class. The ensemble aggregates these scores using either

$$S_{\text{mean}} = \frac{1}{n}\sum_{i=1}^{n} S_i \quad \text{(mean aggregation)} \tag{1}$$

$$S_{\max} = \max_{1\leq i\leq n} S_i \quad \text{(max aggregation)} \tag{2}$$

Score Generation Model We consider an asymmetric Gaussian mixture setting where the class-conditional scores are drawn from two independent but not identically distributed Gaussian random variables. That is, for the negative class (labeled as $-$) and positive class (labeled as $+$), the base learner scores are generated as:

$$S_i^- \overset{\text{iid}}{\sim} \mathcal{N}(0,1), \quad S_i^+ \overset{\text{iid}}{\sim} \mathcal{N}(\mu,\sigma^2), \quad i = 1,\ldots,n, \quad \mu \geq 0,\ \sigma > 0. \tag{3}$$

By fixing the negative-class scores to have zero mean and unit variance, we obtain a simple, two parameter model where the positive-class mean, $\mu > 0$, directly controls class separation, and $\sigma > 0$ controls the variance ratio between the two classes.

2 Analytical Derivations

We derive the Area Under the ROC Curve (AUC) for both aggregation methods. The AUC is defined as the probability that a randomly selected positive instance scores higher

than a randomly selected negative instance: AUC $\triangleq \Pr(S^+ > S^-)$. Throughout, Φ denotes the standard normal CDF and ϕ its density.

2.1 Mean Aggregation

By the properties of independent Gaussian random variables, the mean scores follow:

$$S^-_{\text{mean}} \sim \mathcal{N}\left(0, \frac{1}{n}\right) \text{ and } S^+_{\text{mean}} \sim \mathcal{N}\left(\mu, \frac{\sigma^2}{n}\right), \tag{4}$$

Since the AUC under mean aggregation is given by:

$$\text{AUC}_{\text{mean}} \triangleq \Pr(S^+_{\text{mean}} > S^-_{\text{mean}}) = \Pr\left(S^+_{\text{mean}} - S^-_{\text{mean}} > 0\right), \tag{5}$$

and the difference $S^+_{\text{mean}} - S^-_{\text{mean}}$ is also normally distributed following $\mathcal{N}\left(\mu, \frac{\sigma^2+1}{n}\right)$, we obtain:

$$\text{AUC}_{\text{mean}} = \Phi\left(\frac{\mu}{\sqrt{(\sigma^2+1)/n}}\right) = \Phi\left(\frac{\mu\sqrt{n}}{\sqrt{\sigma^2+1}}\right). \tag{6}$$

This result shows that under mean aggregation the class separation z-score improves with $\sqrt{n}$ (a direct consequence of variance reduction through averaging) and hence AUC_{mean} approaches perfect classification exponentially fast as $n \to \infty$ for any $\mu > 0$. The latter follows from the upper-tail bound of the standard normal distribution (Pollard, 1984):

$$1 - \Phi(z) \;\leq\; \frac{1}{z\sqrt{2\pi}}\, e^{-z^2/2}, \qquad z > 0. \tag{7}$$

Setting $z = \mu\sqrt{n}/\sqrt{\sigma^2+1}$ we obtain the following asymptotic rate for the mis-ranking error probability:

$$1 - \text{AUC}_{\text{mean}} = O\Big(\exp\big\{-\mu^2 n/2(\sigma^2+1)\big\}\Big). \tag{8}$$

2.2 Max Aggregation

For max aggregation, we need to consider the distribution of the maximum of n independent Gaussian random variables. Let $M_n = \max_{1\leq i\leq n} X_i$ where $X_i \sim \mathcal{N}(0,1)$ are independent. The CDF of M_n is:

$$F_{M_n}(x) = \Pr(M_n \leq x) = \prod_{i=1}^{n} \Pr(X_i \leq x) = \Phi(x)^n \tag{9}$$

with density:

$$f_{M_n}(x) = n\phi(x)\Phi(x)^{n-1}. \tag{10}$$

Hence, for our classification setting, $S^-_{\max} = M_n$ with density $f_{M_n}(x)$, and $S^+_{\max} = \mu + \sigma M'_n$ where M'_n is an independent copy of M_n.

The AUC becomes

$$\begin{aligned}
\mathrm{AUC}_{\max} &= \Pr(S^+_{\max} > S^-_{\max}) && (11)\\
&= \int_{-\infty}^{\infty} \Pr(S^+_{\max} > v | S^-_{\max} = v) f_{M_n}(v) dv && (12)\\
&= \int_{-\infty}^{\infty} \Pr(\mu + \sigma M'_n > v) n\phi(v)\Phi(v)^{n-1} dv && (13)\\
&= \int_{-\infty}^{\infty} \Pr\left(M'_n > \frac{v-\mu}{\sigma}\right) n\phi(v)\Phi(v)^{n-1} dv. && (14)
\end{aligned}$$

Since $\Pr(M'_n > x) = 1 - \Phi(x)^n$, we have:

$$\mathrm{AUC}_{\max} = n \int_{-\infty}^{\infty} \phi(v)\Phi(v)^{n-1} \left[1 - \Phi\left(\frac{v-\mu}{\sigma}\right)^n\right] dv. \tag{15}$$

Through integration by parts and simplification, this reduces to

$$\mathrm{AUC}_{\max} = n \int_{-\infty}^{\infty} \underbrace{\phi(v)\,\Phi(v)^{\,n-1}}_{=\frac{\mathrm{d}}{\mathrm{d}v}\Phi(v)^{\,n}} \left[1 - \Phi\Big(\tfrac{v-\mu}{\sigma}\Big)^n\right] \mathrm{d}v \tag{16}$$

$$\xrightarrow{\text{IBP}} \Phi(v)^{\,n}\left[1 - \Phi\Big(\tfrac{v-\mu}{\sigma}\Big)^n\right]\Big|_{-\infty}^{\infty} + n\int_{-\infty}^{\infty} \Phi(v)^{\,n}\,\Phi\Big(\tfrac{v-\mu}{\sigma}\Big)^{\,n-1} \phi\Big(\tfrac{v-\mu}{\sigma}\Big)\,\frac{\mathrm{d}v}{\sigma} \tag{17}$$

$$= n\int_{-\infty}^{\infty} \Phi(v)^{\,n}\,\Phi\Big(\tfrac{v-\mu}{\sigma}\Big)^{\,n-1} \phi\Big(\tfrac{v-\mu}{\sigma}\Big)\,\frac{\mathrm{d}v}{\sigma}. \tag{18}$$

By changing variables, letting $z = \frac{v-\mu}{\sigma}$ we obtain:

$$\mathrm{AUC}_{\max} = n \int_{-\infty}^{\infty} \phi(z)\Phi(z)^{n-1}\Phi(\mu + \sigma z)^n dz. \tag{19}$$

Remark 1. *Although this integral has no elementary closed form, it converges rapidly due to the normal distribution's super-exponential tail decay and hence can be accurately evaluated with standard numerical quadrature (e.g., composite trapezoidal, uniform-grid Newton–Cotes).*

Asymptotic analysis of max vs. mean aggregation. One can show that $\mathrm{AUC}_{\max}$ improves much more slowly compared to $\mathrm{AUC}_{\text{mean}}$ as $n \to \infty$. In particular, for the equal variance case ($\sigma = 1$), we obtain the following sharp asymptotic rate for the mis-ranking error probability under max aggregation.

Proposition 1. *Let $\mu > 0$ and $\sigma = 1$. Then, as the ensemble size $n \to \infty$,*

$$1 - \mathrm{AUC}_{\max}(n) \;=\; \Theta\big(\exp\{-\mu\sqrt{2\log n}\}\big).$$

Proof. The proof follows from standard results in extreme value theory (cf., e.g., de Haan and Ferreira, 2006).

Let $M_n = \max_{1\le i\le n} Z_i$ and $M'_n = \max_{1\le i\le n} Z'_i$, where $\{Z_i\}_{i=1}^n$ and $\{Z'_i\}_{i=1}^n$ are independent i.i.d. samples from $\mathcal{N}(0,1)$. Since $S^-_{\max} = M_n$ and $S^+_{\max} = \mu + M'_n$, we have

$$1 - \mathrm{AUC}_{\max}(n) \;=\; \Pr\big(S^+_{\max} \le S^-_{\max}\big) \;=\; \Pr\big(M_n - M'_n \ge \mu\big).$$

Define the normalizing constants

$$a_n = \sqrt{2\log n}, \qquad b_n = a_n - \frac{\log\log n + \log(4\pi)}{2a_n}.$$

By the Fisher–Tippett–Gnedenko theorem for Gaussian maxima (Hall, 1979; Leadbetter et al., 1983, Thm. 1.5.3) we have

$$a_n(M_n - b_n) \xrightarrow{d} Y, \qquad a_n(M'_n - b_n) \xrightarrow{d} Y',$$

where Y and Y' are independent standard Gumbel random variables. Hence

$$\Pr\big(M_n - M'_n \ge \mu\big) \;=\; \Pr\big(Y - Y' \;\ge\; \mu a_n\big) + o(1).$$

Because $Y - Y'$ follows a standard logistic distribution (Kotz and Nadarajah, 2000, §2.7), for every $x \ge 0$

$$\frac{1}{2}e^{-x} \;\le\; \Pr(Y - Y' \ge x) \;\le\; e^{-x}.$$

Taking $x = \mu a_n$ and noting $a_n = \sqrt{2\log n}$ yields

$$\frac{1}{2}e^{-\mu\sqrt{2\log n}}\big(1 + o(1)\big) \;\le\; 1 - \mathrm{AUC}_{\max}(n) \;\le\; e^{-\mu\sqrt{2\log n}}\big(1 + o(1)\big),$$

establishing the claimed Θ-rate. □

Generalization for $\sigma \geq 1$ (proof omitted). Using similar extreme-value asymptotic results for Gaussian maxima (cf., e.g., Leadbetter et al., 1983; de Haan and Ferreira, 2006; Kotz and Nadarajah, 2000), Proposition 1 can be extended to all fixed $\sigma \geq 1$. Specifically, for any $\mu > 0$ we can obtain:

$$1 - \mathrm{AUC}_{\max}(n) \;=\; \Theta\Big(c_n \cdot e^{-\mu\sqrt{2\log n}}\Big), \qquad c_n = \Big(n^{-2}\sqrt{\log n}\Big)^{\sigma-1}. \tag{20}$$

Setting $\sigma = 1$ in 20 gives $c_n = 1$ and recovers Proposition 1. While the polynomial factor c_n makes the rate strictly *faster* for $\sigma > 1$ than for $\sigma = 1$, the overall decay is still sub-exponential in n, hence much *slower* than the one obtained under mean-aggregation.

Variance-only discrimination. The general form (20), however, reveals an effect unique to max aggregation: for any $\sigma > 1$ and *no* mean shift (i.e., $\mu = 0$),

$$1 - \mathrm{AUC}_{\max}(n) \;=\; \Theta\Big(n^{-2}\sqrt{\log n}\Big)^{\sigma-1} \;\longrightarrow\; 0, \qquad (n \to \infty),$$

so $\mathrm{AUC}_{\max}(n) \to 1$ purely from a variance advantage. By contrast, mean aggregation is insensitive to pure variance differences: when $\mu = 0$, $\mathrm{AUC}_{\mathrm{mean}} = \frac{1}{2}$ for all n.

A simple way to see the phenomenon is via the typical size of Gaussian maxima, using the fact that for large n, $\mathbb{E}[M_n] \approx \sqrt{2\log n}$.

For the positive class under max aggregation we have $S^+_{\max} = \mu + \sigma M'_n$, so its expected value is $\approx \mu + \sigma\sqrt{2\log n}$, whereas for the negative class $S^-_{\max} = M_n \approx \sqrt{2\log n}$. Thus the *class separation* under max aggregation grows like

$$(\text{positive}) - (\text{negative}) \;\approx\; \mu + (\sigma - 1)\sqrt{2\log n}, \tag{21}$$

which diverges as $n \to \infty$ whenever $\sigma > 1$. One can also show that under max aggregation, the standard deviation of the positive and negative class scores is $\sim 1/\sqrt{\log n}$, and hence we have that the *effective* class separation measured in standard–deviation units grows like $\sim \log n$. This allows $\mathrm{AUC}_{\max} \to 1$, even when $\mu = 0$.

By contrast, mean aggregation only exploits symmetric, within class variance reduction without changing the *mean* separation between classes. Therefore, in the extreme case where $\mu = 0$, the class separation remains 0 for all n, yielding $\mathrm{AUC}_{\mathrm{mean}} = \frac{1}{2}$ with no gain from increasing n.

3 Numerical Results

Overview. We evaluate the derived closed–form and integral expressions for $\mathrm{AUC}_{\mathrm{mean}}$ and $\mathrm{AUC}_{\mathrm{max}}$ and summarize the numerical results in Figures 1 and 2. The mean rule uses the analytic AUC in (6), while the max rule is computed via the one–dimensional integral in (19), evaluated with basic quadrature (cf. Remark 1).

Figure 1 shows that as the number n of base learners grows, $\mathrm{AUC}_{\mathrm{mean}}$ climbs to 1 exponentially fast in n (cf. (8)), while $\mathrm{AUC}_{\mathrm{max}}$ improves more slowly with a sub–exponential law (cf. (20)). An important exception arises when the classes differ primarily in variance: for $\sigma > 1$ and small (or zero) mean shift μ, the max rule benefits from *variance–only discrimination* and can outperform averaging, even at sizeable n (right panel, $\mu = 0$, where AUCmax $\to 1$ but AUCmean $= \frac{1}{2}$ for all n).

The performance landscape in Figure 2 makes this tradeoff explicit. Panels (a)–(c) reveal a phase boundary where $\Delta\mathrm{AUC} = \mathrm{AUC}_{\mathrm{max}} - \mathrm{AUC}_{\mathrm{mean}}$ changes sign: the max rule is favored for small μ and large σ, while the mean rule dominates for larger μ and modest σ. Panel (d) tracks the crossing value $\mu^*(\sigma, n)$ at which the two rules tie. Two monotonicities emerge from the growth laws: (i) μ^* *decreases* with n because the mean rule strengthens at the $\sqrt{n}$ rate, and (ii) μ^* *increases* with σ because the extreme–value drift $(\sigma - 1)\sqrt{2 \log n}$ in (21) makes the max rule harder to beat. Consequently, for any fixed $\mu > 0$ the mean rule eventually overtakes the max rule as n grows, but for finite n and sufficiently large σ the max rule can remain superior.

References

Pollard, D. (1984). *Convergence of Stochastic Processes.* Springer Series in Statistics. Springer–Verlag, Berlin. p. 191.

Hall, P. (1979). On the rate of convergence of normal extremes. *Journal of Applied Probability*, 16(2), 433–439.

Kotz, S. & Nadarajah, S. (2000). *Extreme Value Distributions: Theory and Applications.* Imperial College Press.

Leadbetter, M. R., Lindgren, G. & Rootzén, H. (1983). *Extremes and Related Properties of Random Sequences and Processes.* Springer.

de Haan, L. & Ferreira, A. (2006). *Extreme Value Theory: An Introduction.* Springer.

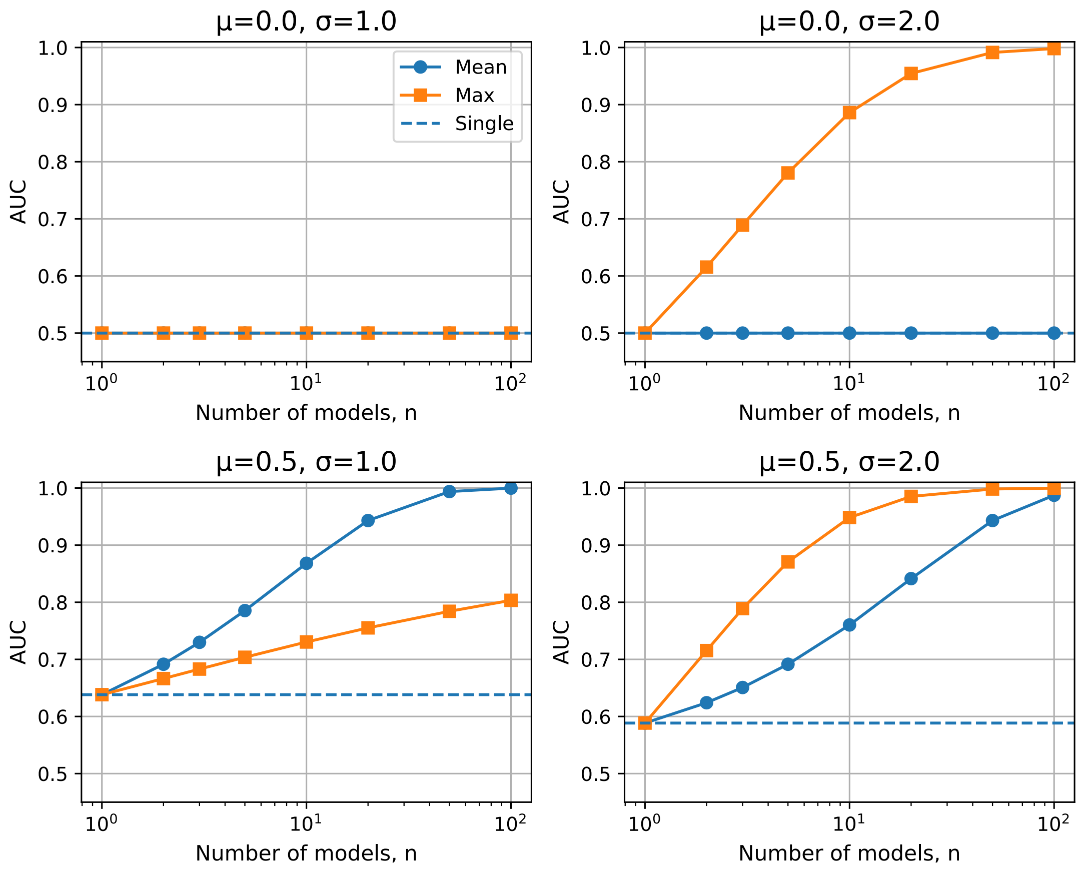

Figure 1: **AUC vs. ensemble size for mean and max aggregation.** Each panel shows $\mathrm{AUC}_{\mathrm{mean}}$ and $\mathrm{AUC}_{\mathrm{max}}$ as a function of n on a log-x scale for the indicated (μ, σ), together with the single-model AUC (dashed). The mean rule follows the closed form (6) and approaches 1 exponentially fast (cf. (8)), whereas the max rule increases more slowly in n (cf. (19) and Proposition 1). For $\sigma > 1$, the max rule can dominate due to variance-only discrimination (right panel, $\mu = 0$), but as μ grows the mean rule can eventually overtake, in line with the scaling laws in §2.2.

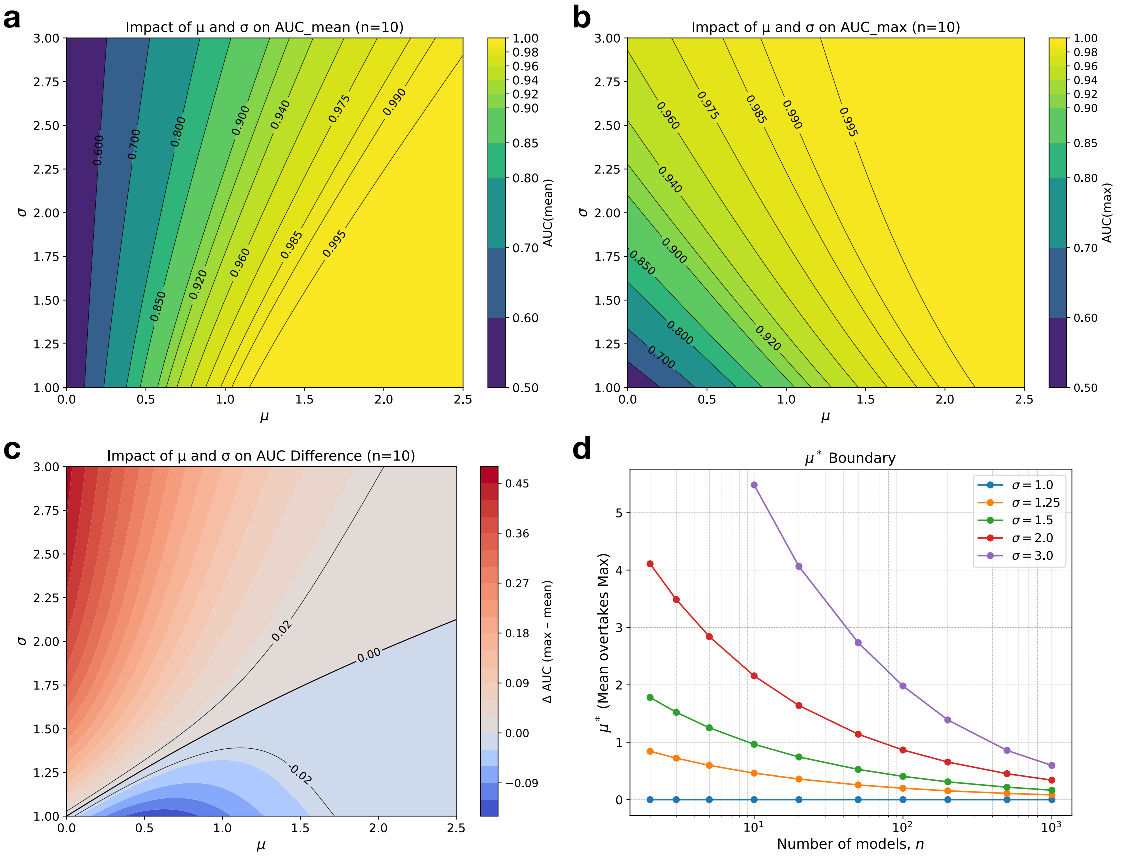

Figure 2: **The performance landscape over** (μ, σ)**.** **(a)** $\mathrm{AUC}_{\mathrm{mean}}$ from (6) for $n = 10$ base models. **(b)** $\mathrm{AUC}_{\mathrm{max}}$ from (19) for $n = 10$ base models. **(c)** Difference $\Delta\mathrm{AUC} = \mathrm{AUC}_{\mathrm{max}} - \mathrm{AUC}_{\mathrm{mean}}$ with zero contour (black) marking the boundary where the rules tie. The red region in (c) (positive Δ) corresponds to regimes where the extreme-value drift favors the max rule, while the blue region (negative Δ) shows where the mean rule is preferable. **(d)** The threshold μ^* where the mean rule overtakes the max rule. For each σ, the curve plots the smallest $\mu^*(\sigma, n)$ such that $\mathrm{AUC}_{\mathrm{mean}}(\mu^*, \sigma, n) = \mathrm{AUC}_{\mathrm{max}}(\mu^*, \sigma, n)$. The boundary decreases with n (the mean rule improves at the $\sqrt{n}$ rate in (6), outpacing the $\sqrt{\log n}$ drift of the max rule; cf. Proposition 1 and (20)), and increases with σ (a larger variance ratio strengthens the max rule).

Supplementary Tables

Supplementary Table 1: ESM base models used for co-distillation

Model	Dataset	Model Size	Reference
ESM-1v<br>(5 models)	UR90	650M	Meier, J. et al. Language models enable zero-shot prediction of the effects of mutations on protein function. 2021.07.09.450648 Preprint at https://doi.org/10.1101/2021.07.09.450648 (2021).<br>Link: facebook/esm1v_t33_650M_UR90S_{1,2,3,4,5}<br>HuggingFace commits: 8bfdb18, 3c1e9e6, 0b00fd1, 443968f, fb2e51c
ESM1b	UR50/S	650M	Rives, A. et al. (ESM1) Biological structure and function emerge from scaling unsupervised learning to 250 million protein sequences. Proc Natl Acad Sci U A 118, (2021).<br>Link: facebook/esm1b_t33_650M_UR50S<br>HuggingFace commit: 7b37824
ESM2 (8M)	UR50 (sample UR90)	8M	Lin, Z. et al. (ESM2) Language models of protein sequences at the scale of evolution enable accurate structure prediction. bioRxiv (2022) doi:10.1101/2022.07.20.500902.<br>Link: facebook/esm2_t6_8M_UR50D<br>HuggingFace commit: c731040
ESM2 (35M)	UR50 (sample UR90)	35M	Lin, Z. et al. (ESM2) Language models of protein sequences at the scale of evolution enable accurate structure prediction. bioRxiv (2022) doi:10.1101/2022.07.20.500902.<br>Link: facebook/esm2_t12_35M_UR50D<br>HuggingFace commit: 6fbf070
ESM2 (150M)	UR50 (sample UR90)	150M	Lin, Z. et al. (ESM2) Language models of protein sequences at the scale of evolution enable accurate structure prediction. bioRxiv (2022) doi:10.1101/2022.07.20.500902.<br>Link: facebook/esm2_t30_150M_UR50D<br>HuggingFace commit: a695f60
ESM2 (650M)	UR50 (sample UR90)	650M	Lin, Z. et al. (ESM2) Language models of protein sequences at the scale of evolution enable accurate structure prediction. bioRxiv (2022) doi:10.1101/2022.07.20.500902.<br>Link: facebook/esm2_t33_650M_UR50D<br>HuggingFace commit: 08e4846
ESM2 (3B)	UR50 (sample UR90)	3B	Lin, Z. et al. (ESM2) Language models of protein sequences at the scale of evolution enable accurate structure prediction. bioRxiv (2022) doi:10.1101/2022.07.20.500902.<br>Link: facebook/esm2_t36_3B_UR50D<br>HuggingFace commit: 476b639
ESM2 (15B)<br>*(only used for co-distillation ablation study)*	UR50 (sample UR90)	15B	Lin, Z. et al. (ESM2) Language models of protein sequences at the scale of evolution enable accurate structure prediction. bioRxiv (2022) doi:10.1101/2022.07.20.500902.<br>Link: facebook/esm2_t48_15B_UR50D<br>HuggingFace commit: 5fbca39

Supplementary Table 2: Training time and VRAM requirements across model sizes

Model	Params	Trainable Parameters	Batch size (sequence)	Peak VRAM (training)	Training time (per epoch)
ESM2 (35M)	35M	3.03M	32	~40GB	7m
ESM2 (650M)	650M	21.4M	12	~80GB	2.25h
ESM2 (3B)	3B	85.4M	4	~77GB	3.5h
ESM3	1.3B	28.3M	4	~60GB	1h

Supplementary Table 3: VESM model inference runtimes and peak VRAM usage

Model	Sequence Length	Peak VRAM (GB)	Throughput (seq/s)	Latency p50 (ms)	Latency p95 (ms)
VESM-35M	short	0.3 +/ 0.0	706.2 +/ 103.3	11.5 +/ 1.7	11.5 +/ 1.7
VESM-35M	medium	0.5 +/ 0.0	359.4 +/ 45.9	22.3 +/ 2.7	23.0 +/ 3.6
VESM-35M	long	1.5 +/ 0.0	155.3 +/ 12.6	51.5 +/ 4.3	52.4 +/ 3.8
VESM-650M	short	2.7 +/ 0.0	88.5 +/ 0.1	90.3 +/ 0.1	90.8 +/ 0.4
VESM-650M	medium	3.0 +/ 0.0	43.8 +/ 0.0	182.7 +/ 0.1	182.9 +/ 0.1
VESM-650M	long	4.1 +/ 0.0	22.2 +/ 0.0	361.1 +/ 0.3	361.6 +/ 0.8
VESM-3B	short	11.2 +/ 0.0	24.3 +/ 0.0	329.0 +/ 0.4	329.8 +/ 0.5
VESM-3B	medium	11.7 +/ 0.0	13.2 +/ 0.0	607.9 +/ 0.5	608.6 +/ 0.2
VESM-3B	long	14.0 +/ 0.0	6.9 +/ 0.0	1160.0 +/ 0.8	1163.2 +/ 3.4
VESM3	short	7.9 +/ 0.0	49.8 +/ 1.4	158.8 +/ 1.7	166.6 +/ 12.7
VESM3	medium	15.5 +/ 0.0	24.6 +/ 0.1	325.6 +/ 0.9	326.2 +/ 1.2
VESM3	long	45.8 +/ 0.0	10.9 +/ 0.0	732.9 +/ 0.5	733.5 +/ 0.2
ESM2 (15B)	short	57.2 +/ 0.0	5.8 +/ 0.0	1380.4 +/ 2.7	1391.1 +/ 15.1
ESM2 (15B)	medium	58.0 +/ 0.0	3.1 +/ 0.0	2589.4 +/ 1.7	2589.7 +/ 1.8
ESM2 (15B)	long	60.4 +/ 0.0	1.5 +/ 0.0	5286.9 +/ 8.5	5288.6 +/ 10.4

Supplementary Table 4: Baseline VEP models

Model	Training Data	Reference
Saprot	sequence, structure	Su, J. et al. SaProt: Protein language modeling with structure-aware vocabulary. bioRxiv (2023) doi:10.1101/2023.10.01.560349, github.com/westlake-repl/SaProt Link: westlake-repl/SaProt_650M_AF2, commit: d11a3d9
ESM3	sequence, structure, function	Hayes, T. et al. Simulating 500 million years of evolution with a language model. 2024.07.01.600583 Preprint at https://doi.org/10.1101/2024.07.01.600583 (2024). Link: EvolutionaryScale/esm3-sm-open-v1, commit: 2feda65

ProSST	sequence, structure	Li, M. et al. ProSST: Protein Language Modeling with Quantized Structure and Disentangled Attention. 2024.04.15.589672 Preprint at https://doi.org/10.1101/2024.04.15.589672 (2024).<br>Link: github.com/ai4protein/ProSST, commit: c05a06f
ProtSSN	sequence, structure	Tan, Y., Zhou, B., Zheng, L., Fan, G. & Hong, L. Semantical and Topological Protein Encoding Toward Enhanced Bioactivity and Thermostability. 2023.12.01.569522 Preprint at https://doi.org/10.1101/2023.12.01.569522 (2023).<br>Link: github.com/ai4protein/ProtSSN, commit: 61e5aa9
RSALOR	structure, MSA	Tsishyn, M., Hermans, P., Pucci, F. & Rooman, M. Residue conservation and solvent accessibility are (almost) all you need for predicting mutational effects in proteins. 2025.02.03.636212 Preprint at https://doi.org/10.1101/2025.02.03.636212 (2025).<br>Link: github.com/3BioCompBio/RSALOR, commit: ea7f1be
PoET	MSA	Jr, T. F. T. & Bepler, T. PoET: A generative model of protein families as sequences-of-sequences. Preprint at https://doi.org/10.48550/arXiv.2306.06156 (2023).<br>Link: github.com/OpenProteinAI/PoET, commit: 9b2239b
TranceptEVE	MSA	Notin, P. et al. TranceptEVE: Combining Family-specific and Family-agnostic Models of Protein Sequences for Improved Fitness Prediction. 2022.12.07.519495 Preprint at https://doi.org/10.1101/2022.12.07.519495 (2022).<br>Link: github.com/OATML-Markslab/ProteinGym, commit: 1f8de97
EVE	MSA	Frazer, J. et al. Disease variant prediction with deep generative models of evolutionary data. Nature 599, 91–95 (2021).<br>Link: github.com/OATML-Markslab/ProteinGym, commit: 1f8de97
DeepSequence	MSA	Riesselman, A. J., Ingraham, J. B. & Marks, D. S. Deep generative models of genetic variation capture the effects of mutations. Nat. Methods 15, 816–822 (2018).<br>Link: github.com/OATML-Markslab/ProteinGym, commit: 1f8de97
EVmutation	MSA	Hopf, T. A. et al. Mutation effects predicted from sequence co-variation. Nat Biotechnol 35, 128–135 (2017).<br>Link: github.com/OATML-Markslab/ProteinGym, commit: 1f8de97
Tranception	MSA	Notin, P. et al. Tranception: Protein Fitness Prediction with Autoregressive Transformers and Inference-time Retrieval. In Proceedings of the 39th International Conference on Machine Learning 16990–17017 (PMLR, 2022).<br>Link: github.com/OATML-Markslab/ProteinGym, commit: 1f8de97
Tranception (NR)	sequence	Notin, P. et al. Tranception: Protein Fitness Prediction with Autoregressive Transformers and Inference-time Retrieval. In Proceedings of the 39th International Conference on Machine Learning 16990–17017 (PMLR, 2022).<br>Link: github.com/OATML-Markslab/ProteinGym, commit: 1f8de97
GEMME	MSA	Laine E, Karami Y, Carbone A. GEMME: A Simple and Fast Global Epistatic Model Predicting Mutational Effects. Mol Biol Evol. 2019 Nov 1;36(11):2604-2619. PMID: 31406981.<br>Link: github.com/OATML-Markslab/ProteinGym, commit: 1f8de97
SIFT	MSA	Ng, P. C. & Henikoff, S. SIFT: Predicting amino acid changes that affect protein function. Nucleic Acids Res 31, 3812–3814 (2003).<br>Link: github.com/OATML-Markslab/ProteinGym, commit: 1f8de97

PROVEAN	MSA	Choi, Y. & Chan, A. P. PROVEAN web server: a tool to predict the functional effect of amino acid substitutions and indels. Bioinformatics 31, 2745–2747 (2015).<br>Link: github.com/OATML-Markslab/ProteinGym, commit: 1f8de97
PrimateAI	population-based	Parry, D. A. et al. PrimateAI-3D outperforms AlphaMissense in real-world cohorts. bioRxiv (2024) doi:10.1101/2024.01.12.24301193.<br>Link: github.com/OATML-Markslab/ProteinGym, commit: 1f8de97
CADD	population-based	Rentzsch, P., Witten, D., Cooper, G. M., Shendure, J. & Kircher, M. CADD: predicting the deleteriousness of variants throughout the human genome. Nucleic Acids Res. 47, D886–D894 (2019).<br>Link: github.com/OATML-Markslab/ProteinGym, commit: 1f8de97
MSA Transformer	MSA	Rao, R. M. et al. MSA Transformer. In Proceedings of the 38th International Conference on Machine Learning (PMLR, 2021).<br>Link: github.com/OATML-Markslab/ProteinGym, commit: 1f8de97
VESPA	sequence	Marquet, C. et al. Embeddings from protein language models predict conservation and variant effects. Hum. Genet. 141, 1629–1647 (2022).<br>Link: github.com/OATML-Markslab/ProteinGym, commit: 1f8de97
VespaG	MSA	Marquet, C., Schlensok, J., Abakarova, M., Rost, B. & Laine, E. VespaG: Expert-guided protein Language Models enable accurate and blazingly fast fitness prediction.<br>Link: github.com/OATML-Markslab/ProteinGym, commit: 1f8de97
FATHMM	sequence	Shihab HA, Gough J, Cooper DN, Stenson PD, Barker GLA, Edwards KJ, et al. Predicting the functional, molecular, and phenotypic consequences of amino acid substitutions using hidden Markov models. Hum Mutat. 2013 Jan;34(1):57–65.<br>Link: github.com/OATML-Markslab/ProteinGym, commit: 1f8de97
DANN	population-based	Quang D, Chen Y, Xie X. DANN: a deep learning approach for annotating the pathogenicity of genetic variants. Bioinformatics. 2015 Mar 1;31(5):761-3. doi: 10.1093/bioinformatics/btu703. Epub 2014 Oct 22. PMID: 25338716; PMCID: PMC4341060.<br>Link: github.com/OATML-Markslab/ProteinGym, commit: 1f8de97
ESM2 (3B)	sequence	Lin, Z. et al. Language models of protein sequences at the scale of evolution enable accurate structure prediction. bioRxiv (2022) doi:10.1101/2022.07.20.500902.<br>Link: facebook/esm2_t36_3B_UR50D, commit: 476b639
ESM2 (15B)	sequence	Lin, Z. et al. Language models of protein sequences at the scale of evolution enable accurate structure prediction. bioRxiv (2022) doi:10.1101/2022.07.20.500902.<br>Link: facebook/esm2_t48_15B_UR50D, commit: 5fbca39
ESM-IF1	structure	Hsu C, Verkuil R, Liu J, Lin Z, Hie B, Sercu T, et al. Learning inverse folding from millions of predicted structures [Internet]. bioRxiv; 2022 [cited 2025 Apr 29]. p. 2022.04.10.487779. Available from: biorxiv.org/content/10.1101/2022.04.10.487779v2<br>Link: katielink/esm_if1_gvp4_t16_142M_UR50, commit: 574b908
ESM-C (300M)	sequence	ESM Team. ESM Cambrian: Revealing the mysteries of proteins with unsupervised learning. https://evolutionaryscale.ai/blog/esm-cambrian (2024).<br>Link: EvolutionaryScale/esmc-300m-2024-12, commit: a19d363

ESM-C (600M)	sequence	ESM Team. ESM Cambrian: Revealing the mysteries of proteins with unsupervised learning. https://evolutionaryscale.ai/blog/esm-cambrian (2024).<br>Link: EvolutionaryScale/esmc-600m-2024-12, commit: d11cc14
ISM	sequence, structure	Ouyang-Zhang, J. et al. Distilling Structural Representations into Protein Sequence Models. 2024.11.08.622579 Preprint at https://doi.org/10.1101/2024.11.08.622579 (2025).<br>Link: jozhang97/ismc-600m-2024-12, commit: 35c2d5a
ProGen2	sequence	Nijkamp, E., Ruffolo, J., Weinstein, E. N., Naik, N. & Madani, A. ProGen2: Exploring the boundaries of protein language models. arXiv [cs.LG] (2022).<br>Link: github.com/OATML-Markslab/ProteinGym, commit: 1f8de97
ProtGPT2	sequence	Ferruz, N., Schmidt, S. & Höcker, B. ProtGPT2 is a deep unsupervised language model for protein design. Nat. Commun. 13, 4348 (2022).<br>Link: github.com/OATML-Markslab/ProteinGym, commit: 1f8de97
CARP	sequence	Yang KK, Fusi N, Lu AX. Convolutions are competitive with transformers for protein sequence pretraining [Internet]. bioRxiv; 2023 [cited 2025 Apr 29]. p. 2022.05.19.492714. Available from: biorxiv.org/content/10.1101/2022.05.19.492714v4<br>Link: github.com/OATML-Markslab/ProteinGym, commit: 1f8de97
MIF	structure	Yang, K. K., Yeh, H. & Zanichelli, N. Masked Inverse Folding with Sequence Transfer for Protein Representation Learning. 2022.05.25.493516 Preprint at https://doi.org/10.1101/2022.05.25.493516 (2023).<br>Link: github.com/OATML-Markslab/ProteinGym, commit: 1f8de97
MULAN	sequence, structure	MULAN: Multimodal Protein Language Model for Sequence and Structure Encoding | bioRxiv. biorxiv.org/content/10.1101/2024.05.30.596565v1.<br>Link: github.com/OATML-Markslab/ProteinGym, commit: 1f8de97
ProteinMPNN	structure	Dauparas, J. et al. Robust deep learning–based protein sequence design using ProteinMPNN. Science (2022).<br>Link: github.com/OATML-Markslab/ProteinGym, commit: 1f8de97
RITA	sequence	Hesslow, D., Zanichelli, N., Notin, P., Poli, I. & Marks, D. RITA: a Study on Scaling Up Generative Protein Sequence Models. Preprint at https://doi.org/10.48550/arXiv.2205.05789 (2022).<br>Link: github.com/OATML-Markslab/ProteinGym, commit: 1f8de97
UniRep	sequence	Alley, E. C., Khimulya, G., Biswas, S., AlQuraishi, M. & Church, G. M. Unified rational protein engineering with sequence-based deep representation learning. Nat. Methods (2019).<br>Link: github.com/OATML-Markslab/ProteinGym, commit: 1f8de97
UniRep (evo)	MSA	Alley, E. C., Khimulya, G., Biswas, S., AlQuraishi, M. & Church, G. M. Unified rational protein engineering with sequence-based deep representation learning. Nat. Methods (2019).<br>Link: github.com/OATML-Markslab/ProteinGym, commit: 1f8de97
Wavenet	MSA	Shin, J.-E. et al. Protein design and variant prediction using autoregressive generative models. Nat. Commun. (2021).<br>Link: github.com/OATML-Markslab/ProteinGym, commit: 1f8de97
